# Supplementary material for: The dual burden of animal and human zoonoses: A systematic review
Source: PLoS Negl Trop Dis. 2022 Oct 14;16(10):e0010540. doi: 10.1371/journal.pntd.0010540 (PMC9605338; doi:10.1371/journal.pntd.0010540)
Supplement: S1 Table — (DOCX) [file pntd.0010540.s001.docx]

### **S1 Table. List of used terms for each electronic search**

**Embase**

| No. | Query | Results |
| --- | --- | --- |
| 1 | 'disability-adjusted life year'/exp OR 'disability-adjusted life year*':ti,ab OR daly:ti,ab OR dalys:ti,ab | **6831** |
| 2 | 'gross national product'/exp OR 'economic aspect'/exp OR 'gross national product*':ti,ab OR  'gross domestic product*’: ti,ab OR gdp:ti,ab OR economic*:ti,ab OR cost*:ti,ab OR  socioeconomic*:ti,ab OR (((animal* OR livestock* OR production* OR monetary OR financial) NEAR/3 loss*):ti,ab) OR (((animal* OR livestock*) NEAR/3 production*):ti,ab) | **2664867** |
| 3 | 'zoonosis'/exp OR zoono*: ti,ab | **765498** |
| 4 | #1 AND #2 AND #3 | **362** |

**Medline**

| No. | Searches | Results |
| --- | --- | --- |
| 1 | ("disability-adjusted life year*" or daly or dalys).ti,ab. | 4704 |
| 2 | exp Gross Domestic Product/ or exp Economics/ or ("gross national product*" or "gross domestic product*" or gdp or economic* or cost* or socioeconomic* or ((animal* or livestock* or production* or monetary or financial) adj3 loss*) or ((animal* or livestock*) adj3 production*)).ti,ab. | 1460356 |
| 3 | exp Zoonoses/ or zoono*.ti,ab. | 41415 |
| 4 | 1 and 2 and 3 | 41 |

**Scopus**

| TITLE-ABS- KEY ("disability-adjusted life year*" OR daly OR dalys) AND TITLE-ABS-KEY ("gross  national product*“OR "gross domestic product* OR gdp OR economic* OR cost* OR  socioeconomic* OR ((animal* OR livestock* OR production* OR monetary OR financial)  W/3 loss*) OR ((animal* OR livestock*) W/3 production*)) AND TITLE-ABS-KEY (zoono*) | 62 |
| --- | --- |

**Web of Science**

| TS= ("disability-adjusted life year*" OR daly OR dalys) AND TS= ("gross national  product*" OR "gross domestic product*" OR gdp OR economic* OR cost* OR  socioeconomic* OR ((animal* OR livestock* OR production* OR monetary OR financial)  NEAR/3 loss*) OR ((animal* OR livestock*) NEAR/3 production*)) AND TS=(zoono*)* | 43 |
| --- | --- |

**Google Scholar**

| Title words | (zoonotic OR zoonosis OR zoonoses) |  |
| --- | --- | --- |
| Keywords | (“disability-adjusted life years” OR daly OR dalys AND (“gross domestic product” OR gdp) | **44** |

|  | **Deduplication** | |
| --- | --- | --- |
|  | **Before** | **After** |
| Embase | 362 | 325 |
| Medline | 41 | 33 |
| Scopus | 62 | 8 |
| Web of Science | 43 | 8 |
| Google Scholar | 44 | 38 |
| **Total** | **552** | **412** |
