# Supplementary material for: The dual burden of animal and human zoonoses: A systematic review
Source: PLoS Negl Trop Dis. 2022 Oct 14;16(10):e0010540. doi: 10.1371/journal.pntd.0010540 (PMC9605338; doi:10.1371/journal.pntd.0010540)
Supplement: S2 Table — (DOCX) [file pntd.0010540.s002.docx]

### **S2 Table. List of papers excluded at the full-text screening, with reasons for exclusion**

| No. | Authors | Title | Reason for exclusion |
| --- | --- | --- | --- |
| 1 | Okello, W. O., Okello, A. L., Inthavong, P., Tiemann, T., Phengsivalouk, A., Devleesschauwer, B., Shaw, A., Allen, J. | Improved methods to capture the total societal benefits of zoonotic disease control: Demonstrating the cost-effectiveness of an integrated control programme for Taenia solium, soil transmitted helminths and classical swine fever in northern Lao PDR | already has zDALYs |
| 2 | Roth, F., Zinsstag, J., Orkhon, D., Chimed-Ochir, G., Hutton, G., Cosivi, O., Carrin, G., Otte, J. | Human health benefits from livestock vaccination for brucellosis: case study | no losses in animals; cost effectiveness study without actual losses in animals; study of economic benefit for proposed mass vaccination in livestock against brucellosis |
| 3 | Ferdous, J., Islam, A., Machalaba, C., Feferholtz, Y., Rahman, M. A., Hagan, E., Berthe, F. C., Daszak, P., Karesh, W. B., Flora, M. S. | Economic burden of rabies and its impact in Bangladesh through a One Health approach | no full text available; conference paper |
| 4 | El Berbri, I., Mahir, W., Fihri, O. F., Petavy, A. F., Dakkak, A., Bouslikhane, M. | Cystic echinococcosis in morocco: Epidemiology, socio-economic impact and control | systematic review; no original data and calculations; losses and DALYs refer to Saadiid et al. |
| 5 | Keita, Z., Gerber, F., Lechenne, M., Thiero, O., Hattendorf, J., Zinsstag, J., Traoré, A., Traoré, A. K. | Burden of rabies in Mali | no losses in animals, only costs for vaccinations against rabies |
| 6 | Larkins, A. J., Reece, J. F., Shaw, A. P. M., Thrusfield, M. V. | An economic case study of the control of dog-mediated rabies by an animal welfare organisation in Jaipur, India | no losses in animals |
| 7 | Saadiid, A., Amarir, F., Filali, H., Thys, S., Rhalem, A., Kirschvink, N., Raes, M., Marcotty, T., Oukessou, M., Duchateau, L., Sahibi, H., Antoine-Moussiaux, N. | The socio-economic burden of cystic echinococcosis in Morocco: A combination of estimation method | already has zDALYs |
| 8 | van Asseldonk, M., van Wagenberg, C. P., Wisselink, H. J. | Break-even analysis of costs for controlling Toxoplasma gondii infections in slaughter pigs via a serological surveillance program in the Netherlands | no losses in animals; break even analysis, only modelled costs for intervention |
| 9 | Pulliam, J. R., Mirelman, A., Khan, S. U., Sazzad, H. M., Hossain, M. J., Paul, R. C., Luby, S. P., Gurley, E. S. | A cost-effectiveness analysis of human and pig vaccination strategies to reduce the burden of Japanese encephalitis in Bangladesh | no full text available |
| 10 | Noguera LP, Rüegg S, Torgerson P. | The burden of zoonoses in Paraguay: A systematic review | already has zDALYs |
