# Supplementary material for: The dual burden of animal and human zoonoses: A systematic review
Source: PLoS Negl Trop Dis. 2022 Oct 14;16(10):e0010540. doi: 10.1371/journal.pntd.0010540 (PMC9605338; doi:10.1371/journal.pntd.0010540)
Supplement: S3 Table — (DOCX) [file pntd.0010540.s003.docx]

### **S3 Table. List of countries included in the rabies studies (at global and continental levels)**

**Worldwide rabies with more than one country in each cluster** (Hampson et al)

| Cluster | Countries |
| --- | --- |
| North Africa | Algeria, Djibouti, Egypt, Eritrea, Libyan Arab Jamahiriya, Morocco, Somalia, Sudan, Tunisia |
| Congo Basin | Angola, Burundi, Central African Republic, Congo, Democratic Republic of the Congo, Equatorial Guinea, Gabon, Guinea, Rwanda |
| West Africa | Benin, Burkina Faso, Cameroon, Cape Verde, Chad, Cote d'Ivoire, Gambia, Ghana, Guinea-Bissau, Liberia, Mali, Mauritania, Niger, Nigeria, São Tomé and Príncipe, Senegal |
| Southern African Development Community (SADC) | Botswana, Comoros, Ethiopia, Kenya, Lesotho, Madagascar, Malawi, Mauritius, Mozambique, Namibia, Seychelles, South Africa, Swaziland, Uganda, United Republic of Tanzania, Zambia, Zimbabwe |
| Andean | Bolivia, Colombia, Ecuador, Peru, Bolivarian Republic of Venezuela |
| Caribbean | Antigua and Barbuda, Bahamas, Barbados, Cuba, Dominican Republic, Grenada, Haiti, Jamaica, Saint Kitts and Nevis, Saint Lucia, Saint Vincent and the Grenadines, Trinidad, and Tobago |
| Central America and Mexico | Belize, Costa Rica, El Salvador, Guatemala, Guyana, Honduras, Mexico, Nicaragua, Panama, Suriname |
| Southern Cone | Argentina, Chile, Paraguay, Uruguay |
| Eastern Europe | Turkey, Albania, Armenia, Belarus, Bosnia and Herzegovina, Bulgaria, Croatia, Cyprus, Czech Republic, Estonia, Greece, Hungary, Latvia, Lithuania, Malta, Poland, Moldova, Romania, Serbia and Montenegro, Slovakia, Slovenia, The former Yugoslav Republic of Macedonia, Ukraine |
| Eurasia | Afghanistan, Kazakhstan, Kyrgyzstan, Mongolia, Russian Federation, Tajikistan, Turkmenistan, Uzbekistan, Azerbaijan, Georgia |
| Middle East | Bahrain, Islamic Republic of Iran, Iraq, Israel, Jordan, Kuwait, Lebanon, Oman, Qatar, Saudi Arabia, Syrian Arab Republic, United Arab Emirates |
| Asia 2 | Cambodia, Democratic People's Republic of Korea, Lao People's Democratic Republic, Myanmar,  Viet Nam |
| Asia 3 | Bangladesh, Bhutan, Nepal, Pakistan |
| Asia 4 | Philippines, Sri Lanka, Thailand |

**Africa and Asia rabies** (Knobel et al.)

| Regions | Countries |
| --- | --- |
| Africa:  all countries in the mainland with Madagascar | Algeria, Angola, Benin, Botswana, Burkina Faso, Burundi, Cameroon, Central African Republic, Chad, Congo, Cote d'Ivoire, Democratic Republic of Congo, Djibouti, Egypt, Equatorial Guinea, Eritrea, Ethiopia, Gabon, Gambia, Ghana, Guinea, Guinea- Bissau, Kenya, Lesotho, Liberia, Libya, Madagascar, Malawi, Mali, Mauritania, Morocco, Mozambique, Namibia, Niger, Nigeria, Rwanda, Senegal, Sierra Leone, Somalia, South Africa, Sudan, Swaziland, Togo, Tunisia, Tanzania, Uganda, Zambia, Zimbabwe |
| Asia:  South – East Asia region and Western Pacific region with Pakistan (defined by WHO) | India, China, Bangladesh, Bhutan, Brunei, Cambodia; Democratic People's Republic of Korea, Indonesia, Laos Peoples Democratic Republic, Malaysia, Mongolia, Myanmar, Nepal, Pakistan, Papua New Guinea, Philippines, Republic of Korea, Sri Lanka, Thailand, Viet Nam |
