# Supplementary material for: The dual burden of animal and human zoonoses: A systematic review
Source: PLoS Negl Trop Dis. 2022 Oct 14;16(10):e0010540. doi: 10.1371/journal.pntd.0010540 (PMC9605338; doi:10.1371/journal.pntd.0010540)
Supplement: S4 Table — (DOCX) [file pntd.0010540.s004.docx]

### **S4 Table. List of papers with zDALY estimates excluded from this systematic review**

| Authors | Zoonotic disease/ pathogen | Year of data source | Country/  Region | DALY | ALE | zDALY | Uncertainty distribution |
| --- | --- | --- | --- | --- | --- | --- | --- |
| Okello, W. O., Okello, A. L., Inthavong, P., Tiemann, T., Phengsivalouk, A., Devleesschauwer, B., Shaw, A., Allen, J. | Neurocysticercosis (T. Solium) | 2014 | Northern Lao PDR | *Taenia solium* 3478 | 13 | 3497 | **----** |
| Saadiid, A., Amarir, F., Filali, H., Thys, S., Rhalem, A., Kirschvink, N., Raes, M., Marcotty, T., Oukessou, M., Duchateau, L., Sahibi, H., Antoine-Moussiaux, N. | Cystic echinococcosis | 2011-2014 | Morocco | 160 (106–238) | 18170 | 18,330 (17,775–19,074) | 95% CI |
| Noguera LP, Rüegg S, Torgerson P. | Babesiosis, brucellosis, campylobacteriosis, chagas, colibacillosis, cryptoporidiosis, cystic echinococcosis,  cysticercosis, dermatophytosis (M. canis),  dioctophymosis, ehrlichiosis, fasciolasis, giardiasis, hantavirus pulmonary syndrome, leishmaniasis, leptospirosis, myaisis, rabies, salmonellosis (non-typhoidal), scabies,  staphylococcosis, streptococcosis,  toxocariasis,  toxoplasmosis, bovine tuberculosis | 2000-2019 | Paraguay | Total: 18,424 (14,859-28,750) | Total: 43,123 (31,501-54,558) | Total: 62,178 (48,696-77,188) | 95% CI |
