# Supplementary material for: The dual burden of animal and human zoonoses: A systematic review
Source: PLoS Negl Trop Dis. 2022 Oct 14;16(10):e0010540. doi: 10.1371/journal.pntd.0010540 (PMC9605338; doi:10.1371/journal.pntd.0010540)
Supplement: S5 Table — (DOCX) [file pntd.0010540.s005.docx]

### **S5 Table. ROBIS**

**Phase 2: Identifying concerns with the review process**

| **DOMAIN 1: STUDY ELIGIBILITY CRITERIA** | |
| --- | --- |
| Describe the study eligibility criteria, any restrictions on eligibility and whether there was evidence that objectives and eligibility criteria were pre-specified: | |
| - 1. Did the review adhere to pre-defined objectives and eligibility criteria? **YES**   The systematic review clearly outlines the objective in the abstract and the last paragraph of the introduction. Exclusion and inclusions criteria are stated both in the systematic review and the study. The authors provided details of eligibility criteria in the *Methods* section.   - 1. Were the eligibility criteria appropriate for the review question? **YES**   The study stated the need to identify studies that quantify the dual burden of zoonoses in humans expressed as DALYs and in animals in terms of monetary losses. Applied eligibility criteria appear to be appropriate for the study.   - 1. Were eligibility criteria unambiguous?  **YES**   The study design is clearly stated.   - 1. Were all restrictions in eligibility criteria based on study characteristics  **YES** appropriate (e.g., date, sample size, study quality, outcomes   measured)?  The eligibility criteria were appropriate for the studies that we included in the systematic review.   - 1. Were any restrictions in eligibility criteria based on sources of **Probably yes** information appropriate (e.g. publication status or format, language,   availability of data)?  The search in electronic databases was not restricted by language. At the full text stage, two papers could not be accessed, thus excluded. | |
| Concerns regarding specification of study eligibility criteria  **LOW**  Rationale for concern: The study has clear research question and objectives. We prespecified the protocol beforehand. The eligibility criteria were adhered to. All signaling questions were answered “YES” or “probably yes”, which raises no concerns regarding the specification of the study eligibility criteria | |
| **DOMAIN 2: IDENTIFICATION AND SELECTION OF STUDIES** | |
| Describe methods of study identification and selection (e.g., number of reviewers involved): | |
| - 1. Did the search include an appropriate range of databases/electronic **YES**   sources for published and unpublished reports?  Embase, Ovid Medline, Scopus, Web of Science and Google Scholar were searched. This can be judged as an appropriate range.   - 1. Were methods additional to database searching used to identify **NO**   relevant reports?  We considered that the five databases that we used for this study were adequate to achieve the objectives of this study.   - 1. Were the terms and structure of the search strategy likely to retrieve **YES**   as many eligible studies as possible?  The full search strategy is reported in the Appendix to the manuscript.   - 1. Were restrictions based on date, publication format, or language. **Probably yes** appropriate?   We added the minimum of restrictions, so we had a higher probability to include the searched studies in this review. The database search itself was not restricted to language or date.   - 1. Were efforts made to minimise error in selection of studies? **YES**   Inclusion assessment was done independently with two reviewers both at the screening and assessing full-text articles. | |
| Concerns regarding methods used to identify and/or select studies **LOW**  Rationale for concern: The reviewers included appropriate range of databases. The used search items were prepared by experienced medical librarian. The were no restrictions on time period nor language. | |
| **DOMAIN 3: DATA COLLECTION AND STUDY APPRAISAL** | |
| Describe methods of data collection, what data were extracted from studies or collected through other means, how risk of bias was assessed (e.g., number of reviewers involved) and the tool used to assess risk of bias: | |
| - 1. Were efforts made to minimise errors in data collection? **YES**   Two reviewers performed data extraction, using agreed format and excel sheet.  3.2 Were sufficient study characteristics available for both review authors  **YES**  and readers to be able to interpret the results?  Detailed study characteristics and results are provided in the manuscript.   - 1. Were all relevant study results collected for use in the synthesis? **YES**   Based on the inclusion and exclusion criteria of this study, all the results collected were relevant.  3.4 Was risk of bias (or methodological quality) formally assessed using **YES** appropriate criteria?  We followed two guidelines, PRISMA guidelines for systematic reviews, and ROBIS tool in order to assess qualitative bias.  3.5 Were efforts made to minimize error in risk of bias assessment? **YES**  Two authors independently assessed the study quality following a protocol and two guidelines (PRISMA and ROBIS tool) | |
| Concerns regarding methods used to collect data and appraise studies  Rationale for concern: **LOW** | |

| **DOMAIN 4: SYNTHESIS AND FINDINGS** |
| --- |
| Describe synthesis methods: |
| - 1. Did the synthesis include all studies that it should? **YES**   PRISMA flowchart illustrates that there were 14 eligible studies. Our synthesis included 14 studies.   - 1. Were all pre-defined analyses reported or departures explained? **YES**   The methods section of the manuscripts addresses all the analyses performed. The authors also provide the script on GitHub to reproduce the study.   - 1. Was the synthesis appropriate given the nature and similarity in **Probably no** the research questions, study designs, and outcomes across   included studies?  The authors did not perform a meta-analysis; however, they justify the reasons for not doing so. The discussion section of the manuscript expands on the qualitative characteristics of the studies included in the synthesis.   - 1. Was between-study variation (heterogeneity) minimal or **No Info** addressed in the synthesis?   No information on statistical heterogeneity, since we do not perform a meta-analysis.   - 1. Were the findings robust, e.g., as demonstrated through funnel **Probably no** plot or sensitivity analyses?   Due to the differences in the included studies and non-comparability we were not able to provide funnel plots. The nature of stochastic methods implies that sensitivity analysis is implicit in the calculations. We performed only a selective sensitivity analysis.   - 1. Were biases in primary studies minimal or addressed in the **Probably yes**   synthesis?  Authors do not assess directly the biases present in the included studies, however in the discussion we outline biases. |
| Concerns regarding the synthesis and findings **LOW**  Rationale for concern: The only concern regarding this domain lies within the fact that we did not assess directly the statistical heterogeneity, however, all the reasons behind these decisions are explained in the manuscript. |

**Phase 3: Judging risk of bias**

| **Domain** | **Concern** | **Rationale for concern** |
| --- | --- | --- |
| 1. Concerns regarding specification of study  eligibility criteria | Low | Almost all signaling questions were answered as “Yes” |
| 2. Concerns regarding methods used to  identify and/or select studies | Low | The screening and full text analysis is clearly described in the methods and involved two reviewers. Both reviewers acted independently. |
| 3. Concerns regarding methods used to  collect data and appraise studies | Low | Two reviewers extracted the data independently. |
| 4. Concerns regarding the synthesis and  findings | Low | Authors did not address heterogeneity in their analysis, because as such the traditional meta-analysis was not performed. However, authors expand on qualitative characteristics of the study and analysis results in the discussion section. |

| **RISK OF BIAS IN THE REVIEW** |
| --- |
| Describe whether conclusions were supported by the evidence: |
| 1. Did the interpretation of findings address all of the concerns **Probably yes**   identified in Domains 1 to 4?  Almost all the signaling questions received “YES” or at least “Probably no”   1. Was the relevance of identified studies to the review's research **YES**   question appropriately considered?   1. Did the reviewers avoid emphasizing results on the basis of their **YES**   statistical significance?  The conclusions in the manuscript reflect all the results. |
| Risk of bias in the review **RISK: LOW**  Rationale for risk: Almos tall the signaling questions raised no concerns in the first three domains and were answered mostly with “yes” or “probably yes.” Only the fourth domain outlined the need to report study variation, but we explain why it is not possible in the discussion. |
