# Supplementary material for: The dual burden of animal and human zoonoses: A systematic review
Source: PLoS Negl Trop Dis. 2022 Oct 14;16(10):e0010540. doi: 10.1371/journal.pntd.0010540 (PMC9605338; doi:10.1371/journal.pntd.0010540)
Supplement: S1 Text — (DOCX) [file pntd.0010540.s007.docx]

### **S1 Text. List of included studies**

1. Knobel DL, Cleaveland S, Coleman PG, et al. Re-evaluating the burden of rabies in Africa and Asia. Bull World Health Organ 2005; 83: 360–8.
2. Budke CM, Deplazes P, Torgerson PR. Global socioeconomic impact of cystic echinococcosis. Emerg Infect Dis 2006; 12: 296–303.
3. Budke CM, Jiamin Q, Qian W, Torgerson PR. Economic effects of echinococcosis in a disease-endemic region of the Tibetan Plateau. Am J Trop Med Hyg 2005; 73: 2–10.
4. Trevisan C, Praet N, Pondja A, et al. Assessment of the social burden of Taenia solium Cysticercosis in Angónia District, Mozambique. Trop Med Int Heal 2013; 18: 109–10.
5. Praet N, Speybroeck N, Manzanedo R, et al. The disease burden of Taenia solium cysticercosis in Cameroon. PLoS Negl Trop Dis 2009; 3. DOI:10.1371/journal.pntd.0000406.
6. Moro PL, Budke CM, Schantz PM, Vasquez J, Santivañez SJ, Villavicencio J. Economic impact of cystic echinococcosis in Peru. PLoS Negl Trop Dis 2011; 5. DOI:10.1371/journal.pntd.0001179.
7. Hampson K, Coudeville L, Lembo T, et al. Estimating the Global Burden of Endemic Canine Rabies. PLoS Negl Trop Dis 2015; 9: 1–20.
8. van Asseldonk MA, Prins J, Bergevoet RH. Economic assessment of Q fever in the Netherlands. Prev Vet Med 2013; 112: 27–34.
9. Trevisan C, Devleesschauwer B, Schmidt V, Winkler AS, Harrison W, Johansen M V. The societal cost of Taenia solium cysticercosis in Tanzania. Acta Trop 2017; 165: 141–54.
10. Shwiff SA, Brown VR, Dao TT, et al. Estimating the economic impact of canine rabies to Viet Nam 2005-2014. PLoS Neglected Trop Dis [electronic Resour 2018; 12: e0006866.
11. Sultanov AA, Abdrakhmanov SK, Abdybekova AM, Karatayev BS, Torgerson PR. Rabies in Kazakhstan. PLoS Neglected Trop Dis [electronic Resour 2016; 10: e0004889.
12. Charypkhan D, Sultanov AA, Ivanov NP, Baramova SA, Taitubayev MK, Torgerson PR. Economic and health burden of brucellosis in Kazakhstan. Zoonoses Public Health 2019; 66: 487–94.
13. Sanhueza JM, Baker MG, Benschop J, Collins-Emerson JM, Wilson PR, Heuer C. Estimation of the burden of leptospirosis in New Zealand. Zoonoses Public Heal 2020; 67: 167–76.
14. Ari Ho, Işlek E, Bilir Mk, ... The monetary impact of zoonotic diseases on society: The Turkish Case Study. Ankara … 2022. <http://vetjournal.ankara.edu.tr/en/pub/auvfd/issue/48904/789598>.
