# Supplementary figures and images for: The dual burden of animal and human zoonoses: A systematic review
Source: PLoS Negl Trop Dis. 2022 Oct 14;16(10):e0010540. doi: 10.1371/journal.pntd.0010540 (PMC9605338; doi:10.1371/journal.pntd.0010540)

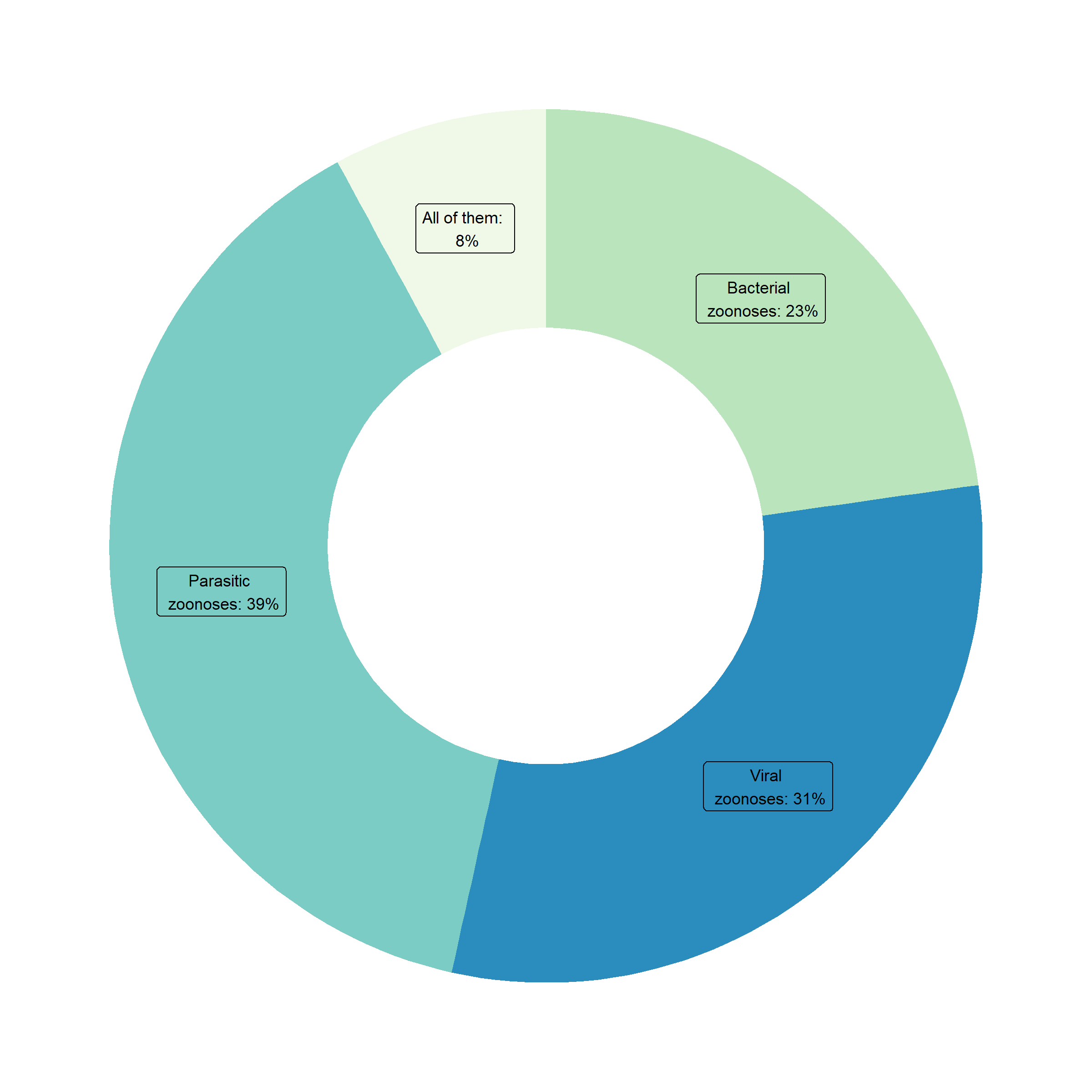

Supplement: S1 Fig — (TIFF) [file pntd.0010540.s008.tiff]

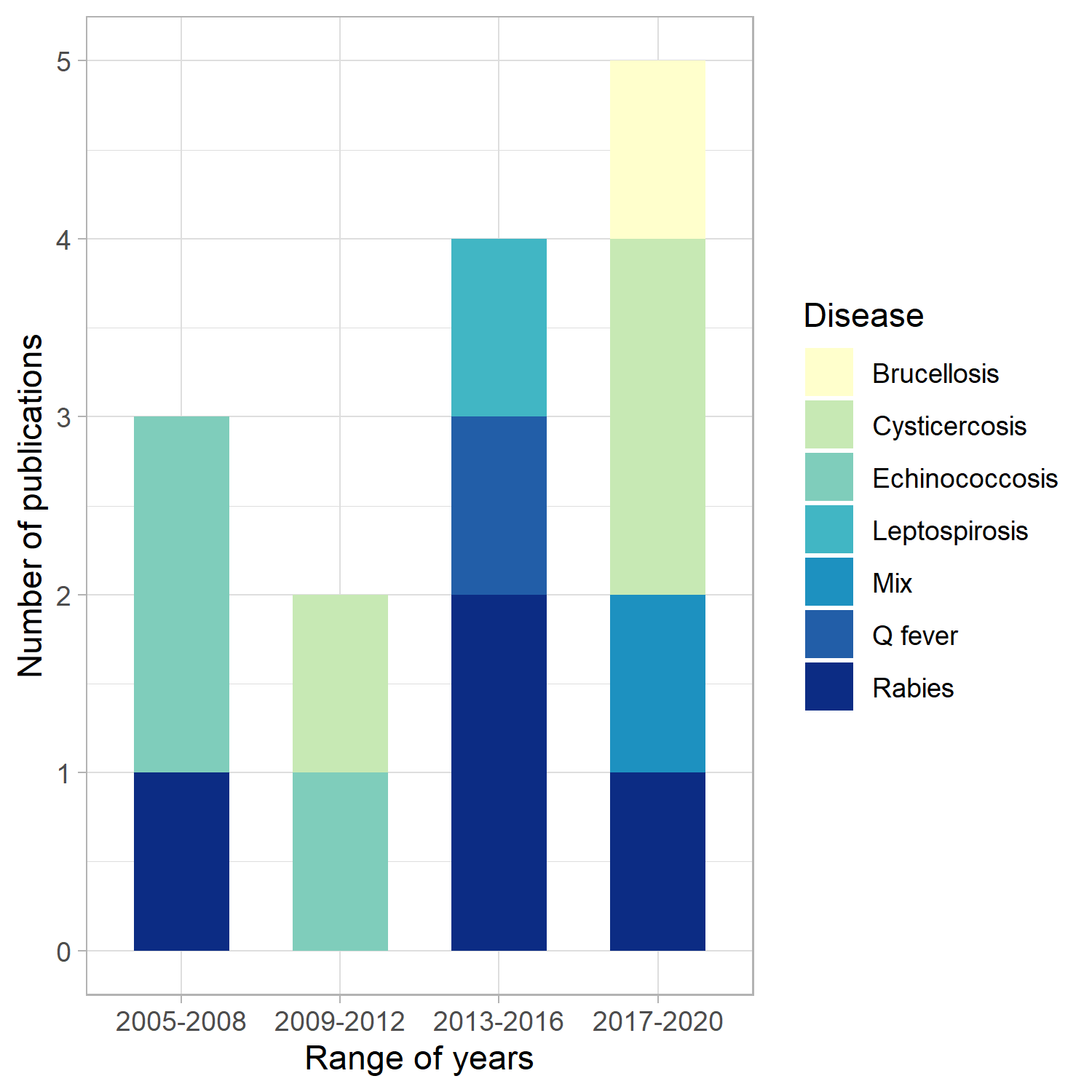

Supplement: S2 Fig — (TIFF) [file pntd.0010540.s009.tiff]

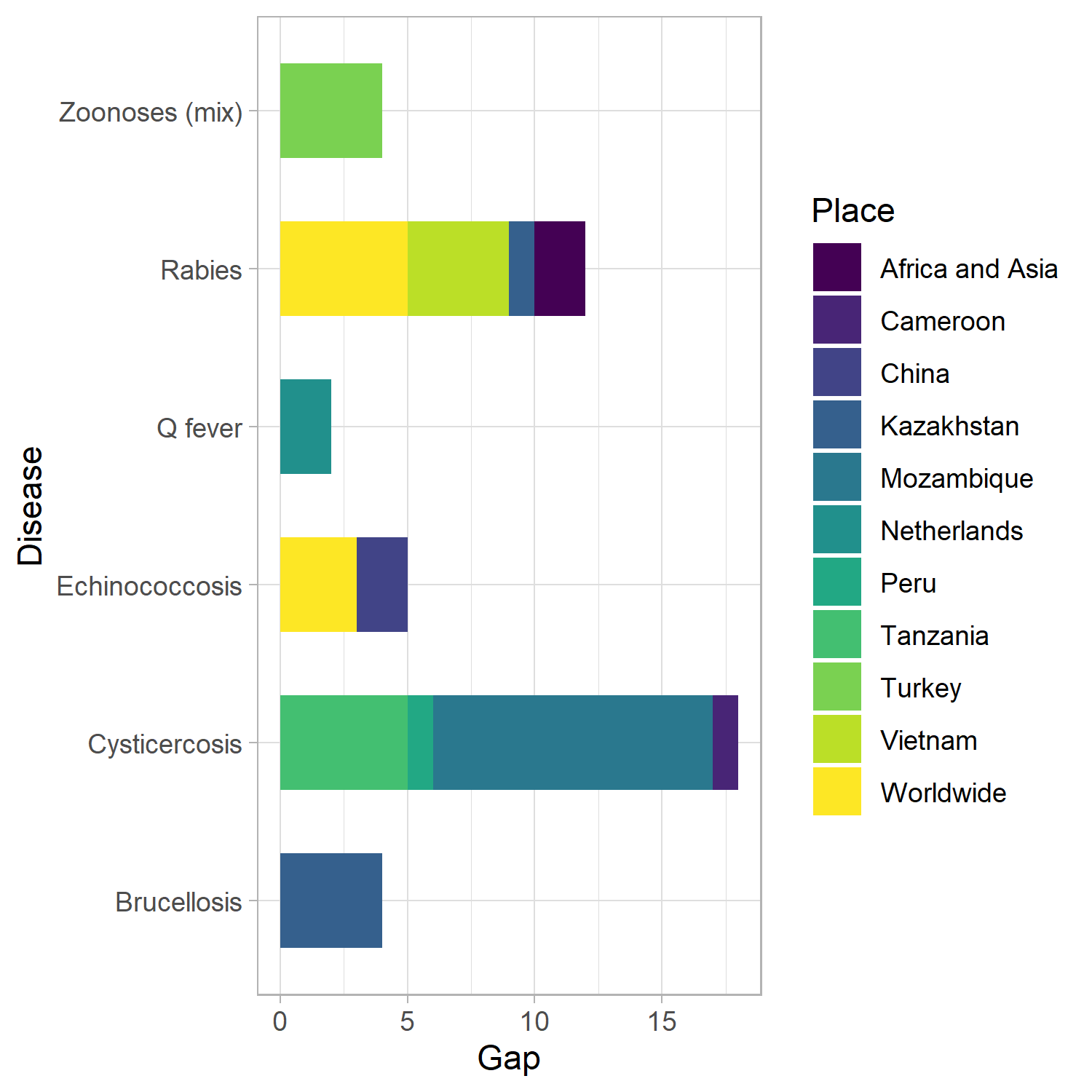

Supplement: S3 Fig — The same happened with human and animal data. (TIFF) [file pntd.0010540.s010.tiff]
