# Supplementary material for: The dual burden of animal and human zoonoses: A systematic review
Source: PLoS Negl Trop Dis. 2022 Oct 14;16(10):e0010540. doi: 10.1371/journal.pntd.0010540 (PMC9605338; doi:10.1371/journal.pntd.0010540)
Supplement: Attachment — Submitted filename: PNTD-D-22-00699.pdf [file pntd.0010540.s012.pdf]

PLOS Neglected Tropical Diseases

The dual burden of animal and human zoonoses: a systematic review

--Manuscript Draft--

**Manuscript Number:**	PNTD-D-22-00699
**Full Title:**	The dual burden of animal and human zoonoses: a systematic review
**Short Title:**	The dual burden of animal and human zoonoses
**Article Type:**	Research Article
**Keywords:**	zoonosis; dual burden; DALY; zDALY; review
**Abstract:**	Background<br><br>Zoonoses can cause a substantial burden on both human and animal health. Globally, estimates of the dual (human and animal) burden of zoonoses are scarce. Therefore, this study aims to quantify the dual burden of zoonoses using a comparable metric, “zoonosis Disability Adjusted Life Years” (zDALY).<br><br>Methodology/Principal Findings<br><br>We systematically reviewed studies that quantify in the same article zoonoses in animals, through monetary losses, and in humans in terms of Disability Adjusted Life Years (DALYs). We searched EMBASE, Web of Science, Scopus, PubMed, and Google Scholar. We excluded articles that did not provide the data to estimate the zDALY or those for which full text was not available. This study was registered at PROSPERO, CRD42022313081.<br><br>Conclusions/Significance<br><br>We identified 512 potentially eligible records. After deduplication and screening of the title and abstract, 23 records were assessed for full-text review. Fourteen studies were included in this systematic review. The data contains estimates from 10 countries, a study at continental level (Asia and Africa), and 2 studies on a global scale.
**Additional Information:**	
**Question**	**Response**
**Financial Disclosure**<br><br>Enter a financial disclosure statement that describes the sources of funding for the work included in this submission. Review the submission guidelines for detailed requirements. View published research articles from *PLOS NTDs* for specific examples.<br><br>This statement is required for submission and **will appear in the published article** if the submission is accepted. Please make sure it is accurate.	The study was partially funded by “BECAL” (LPN) and the Section of Epidemiology, Vetsuisse faculty (UZH) (PT).<br><br>BECAL had no role in study design, data collection and analysis, decision to publish, or preparation of the manuscript.

Powered by Editorial Manager® and ProduXion Manager® from Aries Systems Corporation

**Unfunded studies**<br><br>Enter: *The author(s) received no specific funding for this work.*<br><br>**Funded studies**<br><br>Enter a statement with the following details:<br>• Initials of the authors who received each award<br>• Grant numbers awarded to each author<br>• The full name of each funder<br>• URL of each funder website<br>• Did the sponsors or funders play any role in the study design, data collection and analysis, decision to publish, or preparation of the manuscript?<br>• **NO** - Include this sentence at the end of your statement: *The funders had no role in study design, data collection and analysis, decision to publish, or preparation of the manuscript.*<br>• **YES** - Specify the role(s) played.<br><br>* typeset	
**Competing Interests**<br><br>Use the instructions below to enter a competing interest statement for this submission. On behalf of all authors, disclose any competing interests that could be perceived to bias this work—acknowledging all financial support and any other relevant financial or non-financial competing interests.<br><br>This statement **will appear in the published article** if the submission is accepted. Please make sure it is accurate. View published research articles from *PLOS NTDs* for specific examples.	The authors have declared that no competing interests exist

**NO authors have competing interests**<br><br>Enter: *The authors have declared that no competing interests exist.*<br><br>**Authors with competing interests**<br><br>Enter competing interest details beginning with this statement:<br><br>*I have read the journal's policy and the authors of this manuscript have the following competing interests: [insert competing interests here]*<br><br>* typeset<br><br>This statement is required for submission and **will appear in the published article** if the submission is accepted. Please make sure it is accurate and that any funding sources listed in your Funding Information later in the submission form are also declared in your Financial Disclosure statement.	
**Data Availability**<br><br>Authors are required to make all data underlying the findings described fully available, without restriction, and from the time of publication. PLOS allows rare exceptions to address legal and ethical concerns. See the PLOS Data Policy and FAQ for detailed information.	Yes - all data are fully available without restriction

Powered by Editorial Manager® and ProduXion Manager® from Aries Systems Corporation

A Data Availability Statement describing where the data can be found is required at submission. Your answers to this question constitute the Data Availability Statement and **will be published in the article**, if accepted.<br><br>**Important:** Stating 'data available on request from the author' is not sufficient. If your data are only available upon request, select 'No' for the first question and explain your exceptional situation in the text box.<br><br>Do the authors confirm that all data underlying the findings described in their manuscript are fully available without restriction?	
**Describe where the data may be found in full sentences. If you are copying our sample text, replace any instances of XXX with the appropriate details.**<br><br>• If the data are **held or will be held in a public repository**, include URLs, accession numbers or DOIs. If this information will only be available after acceptance, indicate this by ticking the box below. For example: *All XXX files are available from the XXX database (accession number(s) XXX, XXX.*).<br>• If the data are all contained **within the manuscript and/or Supporting Information files**, enter the following: *All relevant data are within the manuscript and its Supporting Information files.*<br>• If neither of these applies but you are able to provide **details of access elsewhere**, with or without limitations, please do so. For example:<br><br>*Data cannot be shared publicly because*	All relevant data are within the manuscript and its Supporting Information files. Scripts are available at https://github.com/LizPNZ/Dual-burden-of-zoonosis

of [XXX]. Data are available from the XXX Institutional Data Access / Ethics Committee (contact via XXX) for researchers who meet the criteria for access to confidential data.<br><br>*The data underlying the results presented in the study are available from (include the name of the third party and contact information or URL).*<br>• This text is appropriate if the data are owned by a third party and authors do not have permission to share the data.<br><br>* typeset	
Additional data availability information:	

Manuscript

Click here to access/download;Manuscript;Dual burden of zoonoses_systematic review_Plos NTD.docx

The dual burden of animal and human zoonoses: a systematic review

Liz P. Noguera, [1, 2¶] Duriya Charypkhan [1, 2¶], Sonja Hartnack[1], Paul R. Torgerson[1], Simon R. Rüegg[1]

[1] Section of Epidemiology, Vetsuisse Faculty, University of Zürich, Zürich, Switzerland

[2] Epidemiology and Biostatistics, Life Science Zurich Graduate School, University of Zurich, Zurich, Switzerland

lizpaola.noguerazayas@uzh.ch, lpnogal@gmail.com

duriya.charypkhan@uzh.ch, charypkhan1@gmail.com

Abstract

Background

Zoonoses can cause a substantial burden on both human and animal health. Globally, estimates of the dual (human and animal) burden of zoonoses are scarce. Therefore, this study aims to quantify the dual burden of zoonoses using a comparable metric, "zoonosis Disability Adjusted Life Years" (zDALY).

Methodology/Principal Findings

Comment: you are talking about methods in this paragraph, or findings? your text tells it's about methods and your subtitle says about both. please clarify it. It's better to separate these two parts. On the other hand you wrote your finding in conclusion part.

We systematically reviewed studies that quantify in the same article zoonoses in animals, through monetary losses, and in humans in terms of Disability Adjusted Life Years (DALYs). We searched EMBASE, Web of Science, Scopus, PubMed, and Google Scholar. We excluded articles that did not provide the data to estimate the zDALY or those for which full text was not available. This study was registered at PROSPERO, CRD42022313081.

Conclusions/Significance

We identified 512 potentially eligible records. After deduplication and screening of the title and abstract, 23 records were assessed for full-text review. Fourteen studies were included in this systematic review. The data contains estimates from 10 countries, a study at continental level (Asia and Africa), and 2 studies on a global scale.

The total burden from these 14 studies in zDALYs is 11,015,438 (CI 95%: 6,235,971-15,806,100), most of which is attributable to rabies and echinococcosis worldwide, including the Animal Loss Equivalent (ALE) is 4,936,233 (CI 95%: 3,512,616-6,357,435). These results are based on ten zoonotic diseases (rabies, echinococcosis, cysticercosis, brucellosis, leptospirosis, anthrax, Q-fever, CCHF, tularemia, and toxoplasmosis) which had the biggest impact on the public health sector.

Author Summary

Zoonoses impact humans and animals in several ways. Unfortunately, the burden of zoonoses is usually not characterized and quantified through integrated human and animal metrics. Our study is the first systematic review to assess the dual burden of zoonotic diseases in humans and animals globally. In the considered set of human and animal burden of zoonoses, 45% of the zDALY was due to animal disease. Therefore, metrics encompassing both burdens are likely to change decision-making regarding the prevention and control of zoonoses. Implementing a One Health approach will require the application of such metrics. We believe that quantification of the dual burden of the diseases is a key to improving zoonosis prioritization decision-making, and resource

allocation. This study outlines the need for integrated studies on zoonoses and reporting of data with a comparable metric.

Introduction

Zoonoses are diseases that can be transmitted directly or indirectly from animals to humans (and vice versa, hence anthroponoses). Around 6 in 10 human infections are zoonotic [1]. In the human population, early detection of zoonoses prevents loss of life, well-being, money, time, and productivity. By definition, zoonoses harm domestic animals and may threaten wildlife [2]. Zoonotic diseases also incur financial costs, including those caused by losses to humans, animals, and the environment. Integrated surveillance in animals can provide significant benefits, including knowledge generation. The additional economic benefit of zoonoses surveillance might help decide how much data integration is sought, impacting surveillance types, diseases, and geographical settings. Recent pandemics have highlighted the need for surveillance systems for zoonotic events, and the need for better communication across the human-animal-ecosystems continuum.[3] Because human, animal, and ecosystem health are intimately related, surveillance should be organized in an integrated way [4]. This allows for a comprehensive risk assessment and the design of appropriate responses [5].

The business case for a One Health (OH) approach to mitigation of zoonoses has been presented as a framework [6] which includes the creation of one health surveillance and response programs for future emerging diseases. Animal health surveillance data can be used to inform public health messaging, control measures along the food chain, and establish public health surveillance if a pathogen is present in the human population and public health action is required.

In general, the impact of zoonotic diseases on the human population is measured by financial cost, mortality, morbidity, or other indicators known as disease burden [7]. The specific burden of a disease on humans can be quantified using the Disability Adjusted Life Years (DALY).[8] Methods that estimate the human disease burden in monetary terms include costs associated with the diagnostics and treatment of the disease, the statistical value of a human life, costs related to the loss of productivity or loss of income in humans.

The direct impact of animal disease is studied using various economic models. For example, the burden of diseases can be quantified through the money spent on the disease intervention programs, or money accounted for the loss of animal productivity (less milk/meat yield, etc.). The challenge of economic analysis in a OH context is that the boundaries of the system for which costs and benefits incur can be extended or restricted arbitrarily and hence alternative economic models are needed.

A pragmatic approach to consider the combined burden on human and animal health has been proposed as "*zoonosis Disability Adjusted Life Years*" (zDALYs) [9]. The zDALYs extends the DALY framework to domestic animals. The idea behind this indicator is that the animal burden estimated as monetary losses can be converted to Animal Loss Equivalents (ALE). The ALE is basically a metric that reflects the time trade-off for human life years to "replace" the animal loss, e.g., it is the amount of time that a farmer would need to spend to recover the losses.

Despite the availability of data on the zoonosis burden in humans and animals regarding monetary and societal costs separately, only a few studies have estimated the dual burden in animals and

humans [10–12]. We conducted a systematic review of the literature focusing on socio-economic burden of zoonoses worldwide and estimated the zDALYs of such studies.

Methods

Search strategy and selection criteria

You should refer to supplementary

We followed the guidelines for "Preferred Reporting Items for Systematic reviews and Meta-Analyses (PRISMA) [13]. A medical librarian assisted in the development of the search syntax.

We searched electronic academic databases (Embase, Ovid Medline, Scopus, Web of Science) and internet search engines (Google Scholar) for observational epidemiological studies on, at least, a zoonotic disease that includes human disease burden in DALYs and animal disease burden expressed in monetary terms. We included all peer-reviewed studies from an unrestricted period until November 2021. We excluded non-observational epidemiological studies such as experimental studies (e.g., only molecular biology studies), clinical cases, scientific correspondence, or mathematical models without data on the burden of zoonoses.

The data sources and search terms with results are provided in the supporting information (pp 1-2).

Data extraction

According to the eligibility criteria stated above, the identified titles and abstracts were independently reviewed by two reviewers (LPN and DC). Then, DC and LPN independently assessed the full texts of the included papers and documented the reasons for exclusions. The eligibility disagreements were resolved by group discussion.

The data were independently extracted, and double entered into a Microsoft Excel spreadsheet by the two reviewers. For each study, the size of human and animal populations, diseases, DALYs, and associated animal losses were extracted.

Data analysis

We estimated the Animal Loss Equivalents (ALE) of each finding to calculate the zoonosis Disability Adjusted Life Years (zDALY). We divided the annual monetary value of animal health losses by the Gross National Income (GNI) per capita in US$ at the period of the study. The GNIs were obtained from World Bank Open Data. For the economic losses that were in a different currency than the US$, we converted it into the US$ at the year of the study using a historical currency converter [14].

ALE = annual monetary value of animal health losses/ GNI per capita in US$ at the period of the study

We computed the zDALY, adding the DALY of the findings to the ALE that we estimated.

$$zDALY = DALY + ALE$$

To account for the uncertainty of all estimates, we generated random numbers between the lower and upper bounds of the distributions from the previous studies. We set 100,000 iterations for each estimation. According to the original studies, we reported the 50, 2.5, and 97.5 percentiles of the estimates, and 50, 5, 95 percentiles. We have also kept the terms that previous studies used to express uncertainty.

We performed the analyses in R 4.1.3. Scripts are available at https://github.com/LizPNZ/Dual-burden-of-zoonosis

The stochastic approaches used to calculate zDALYs imply that sensitivity is included in the calculations.

We estimated ALEs and zDALYs for all countries with available data over the study period. We reported bias qualitatively through the ROBIS tool.[15] The ROBIS tool encompasses three phases, the first being optional, as it assesses the relevance of the review and the target question. We considered Phase 1 redundant because its questions are a repetition of the inclusion criteria already described in the protocol and methodology. Phase 2 includes the identification of concerns with the review process, and Phase 3, the judgment of risk of bias.

This study is registered at PROSPERO, CRD42022313081.

Results

why result doesn't referred to flow diagram?

We identified 552 articles through electronic database searches (Figure 1). After removing 140 duplicates, 412 articles were screened for titles and abstracts. The full texts of 23 articles were reviewed and 9 were excluded at this stage. Thus, 14 articles are included in this review (Table 1, supporting information, p 5). Common reasons for exclusion at the full-text screening stage were no relevant data or the absence of data on animal monetary losses, DALYs in humans, or absence of full-text. The list of articles excluded at the full-text stage with the brief reasons for exclusion can be found in supporting information p 3.

Table 1: Findings in the dual burden of zoonoses (ordered by ascending year of the data source

Authors	Period of data source	Zoonotic disease/ pathogen	Country/ Region	DALY	Uncertainty	Animal species	Animal loss
Knobel et al.[16]	Human data: 1996-2000, 2003 Livestock cost: 2002	Rabies	Africa and Asia	**Africa:** 747,918 (217,954-1,449,114)<br><br>**Asia:** 1,039,119 (302,324-1,983,646)<br><br>**Total without PEP:** 9,504,237 (4,848,684-15,264,050)<br><br>**Total:** 1787886 (799615-2984109)	90 % CI	Livestock	**Africa:** US$ 1.7 (1.5–1.9)<br>**Asia:** US$ 10.5 (9.4–11.8)<br>**Total:** US$ 12.3 (11–13.7)<br><br>(All values in million dollars)
Budke et al.[17]	1996-2003	Cystic echinococcosis	Worldwide	**Unadjusted:** 285,407 (218,515–366,133)<br><br>**Adjusted for underreporting:** 1,009,662 (862,119–1,175,654)	95 % CI	Livestock	**Unadjusted:** US$ 1,249,866,660 (942,356,157–1,622,045,957)<br><br>**Adjusted for underreporting:** US$ 2,190,132,464 (1,572,373,055–2,951,409,989)

Budke et al.[18]	Human data: 2001-2003<br>Animal data: 1980, 1997	Echinococcosis	China (Shiqu County)	1100	95 % CI (For animal loss estimation)	Livestock (calves, yaks, meat)	**Total losses** (excluding losses in calf production, carcass weight, and yak hide): US$ 278,292 (240,829–318,249)<br><br>**Total losses** (including losses in calf production, carcass weight, and yak hide): US$ 439,734 (384,342–498,447)
Trevisan et al.[19]	2007	Cysticercosis *(Taenia solium*)	Mozambique (Angónia district)	2003 (1433–2762)	95 % UI	Pigs	US$ 22,282 (12,315–35,647)
Praet et al.[20]	2008	Cysticercosis (*Taenia solium*)	Cameroon	45,838 (14,108–103,469)	95 % CR	Pigs	€ 478,844 (369,587–601,325)
Moro et al.[20]	2010	Cystic echinococcosis	Peru	1,139 (861–1,489)	95 % CI	Livestock	US$ 3846754 (2,676,181–4,911,383)

Hampson et al.[21]	2010	Rabies	Worldwide	3,714,333 (1,316,000–10,519,000)	95 % CI	Livestock	**Total:** 129.55
			Asia 2	357,015 (80,000–655,000)			**Asia 2:** 2.073
			Asia 3	160,801 (75,000–853,000)			**Asia 3:** 0.564
			Asia 4	16,521 (10,000–83,000)			**Asia 4:** 11.248
			China	374,851 (60,000–674,000)			**China:** 4.235
			India	1,301,865 (377,000–3,436,000)			**India:** 9.050
			Indonesia	12,311 (12,000–198,000)			**Indonesia:** 6.384
			North Africa	123,074 (38,000–467,000)			**North Africa:** 2.756

			Congo Basin	449,382 (244,000–1,031,000)			**Congo Basin:** 0.481
			West Africa	375,023 (206,000–971,000)			**West Africa:** 6.684
			SADC	398,164 (157,000–1,713,000)			**SADC:** 4.600
			Andean	1,582 (0–4000)			**Andean:** 10.753
			Brazil	1,023 (0–2000)			**Brazil:** 16.620
			Caribbean	8,581 (4000–17,000)			**Caribbean:** 2.575
			Central America	495 (0–3000)			**Central America:** 31.308
			Southern Cone	270 (0–1000)			**Southern Cone:** 4.710

			Eastern Europe<br>Eurasia<br>Middle East	1,948 (0–5000)<br>117,116 (46,000–368,000)<br>14,310 (6000–39,000)			**Eastern Europe:** 10.460<br>**Eurasia:** 4.451<br>**Middle East:** 0.592<br>(In thousands of US$)
van Asseldonk et al.[22]	2007-2011	Q fever	Netherlands	2462	----	Goats	**Loss culling milk goat:** € 300 /case<br>**Loss breeding prohibition:** € 250/ goat<br>**Total:** € 0.03 Million
Trevisan et al.[23]	2012	Cysticercosis (*Taenia solium*)	Tanzania	31,863 (9136–72,078)	95 % UI	Pigs	US$ 2,800,000 (1,100,000–5,400,000)
Shwiff et al.[24]	2005-2014	Rabies	Viet Nam	12,339	----	Livestock	US$ 10,344,223
Sultanov et al.[25]	2003-2015	Rabies	Kazakhstan	**Total:** 454 (339–593) Without **PEP:** 7827 (4746–12,074)	95 % CI	Livestock (cattle, sheep,	US$ 5,400,000 (4,000,000 – 7,100,000)

						horses and camels)	
Charypkhan et al.[26]	2006-2015	Brucellosis	Kazakhstan	713	----	Cattle, sheep	US$ 21,316,800
Sanhueza et al.[27]	2013-2019	Leptospirosis	New Zealand	**At risk of leptospirosis:** 14.07 (1.86–80.73)<br><br>**Not at risk of leptospirosis:** 3.69 (0.49–21.20)<br><br>**Total:** 17.76 (2.35–101.93)	95% PI	Beef cattle, sheep and deer.	US$ 7.92 (3.75–15.48) million
Ari et al.[28]	2016-2018	Brucella, Anthrax, Tularemia, CCHF, Rabies, Cystic Echinococcosis, Toxoplasmosis	Turkey	**Total:** 1782<br>**Brucella:** 1068<br>**Anthrax:** 50<br>**Tularemia:** 1<br>**CCHF:** 505<br>**Rabies:** 113<br>**Cystic Echinococcosis:** 24<br>**Toxoplasmosis:** 21	----	Livestock (large and small ruminants)	**Total loss in 2016:** US$ 213,674,967<br>**Total loss in 2017:** US$ 263,105,316<br>**Total loss in 2018:** US$ 336,313,908<br><br>**Mean of total loss:** US$ 271,031,397

Asia 2: Cambodia, Myanmar, Laos, Vietnam, and Democratic People's Republic of Korea; Asia 3: Bhutan, Nepal, Bangladesh, Pakistan (Himalayan region); Asia 4: Philippines, Sri Lanka, Thailand; SADC: countries in the Southern African Development Community; Eurasia: Afghanistan, Kazakhstan, Kyrgyzstan, Mongolia, the Russian Federation, Turkmenistan, Tajikistan, and Uzbekistan. More information in the supporting information pp 6-7.

CI: Confidence Interval, UI: Uncertainty Interval, CR: Confidence Region, PI: Prediction Interval

PEP: post-exposure prophylaxis

Figure 1: Literature search and article inclusion

Publications on zoonoses considering human and animal populations that met the inclusion criteria started in 2005. Most reported zoonoses were parasitic, whereas no fungal zoonosis was reported. The most frequently reported zoonoses were rabies, and food-borne diseases such as cystic echinococcosis, and cysticercosis.

The studies considered mainly low- and middle-income countries, except for the Netherlands and New Zealand. Only two studies on rabies and cystic echinococcosis were on a global scale, and one study on rabies in two continents: Africa, and Asia (Figure 2). The preferred currency to measure the economic loss was the U.S dollar for 12 articles, and the euro for studies in Cameroon and the Netherlands.

Figure 2: Zoonoses studied in humans and animals with their year of publication by income countries

All studies performed their assessment of the monetary impact of the disease. In humans, it comprises the costs associated with direct treatment of the medical condition and indirect costs associated with for e.g., transportation. In animals, it was costs associated with lost productivity, organ condemnation, or death.

Ten articles used stochastic methods for their estimations, expressing their uncertainty in a 95% Confidence Interval (CI), Uncertainty Interval (UI), Confidence Region (CR), Prediction Interval (PI), and one with a 90% CI (Table 2).

Table 2: Estimates of the dual burden of zoonoses.

Zoonotic disease/ pathogen	Year	Country/ Region	DALY	ALE	zDALY	Uncertainty and distribution
**Rabies (*Lyssavirus*)**	Human data: 1996-2000, 2003<br><br>Livestock cost: 2002	Africa and Asia	**Africa:** 835,380 (281,198–1,387,050)<br><br>**Asia:** 1141077 (3844311– 1898325)<br><br>**Total:** 1,882,387 (907,507–2,874,205)<br><br>**Total without PEP:** 10,068,537 (5,373,433–14,747,882)	**Africa:** 1858 (1661–2055)<br><br>**Asia:** 4157 (3733 –4580)<br><br>**Total:** 7334 (6612–8055)	**Africa:** 837,158 (283,087–1,388,963)<br><br>**Asia:** 1,145,287 (388,592– 1,902,310)<br><br>**Total:** 1,889,928 (914,795–2,881,607)<br><br>**Total without PEP:** 10,075,831 (5,380,459–14,755,386)	90 % CI Uniform distribution
**Cystic echinococcosis(*E. granulosus*)**	1996-2003	Worldwide	**Unadjusted:**<br>292,111 (222,377–362,385)<br><br>**Adjusted for underreporting:**<br>1,019,530 (869,875–1,167,877)	**Unadjusted:** 2,782,397 (2,084,548–3,489,591)<br><br>**Adjusted for underreporting:**<br>4,916,173(3,495,999–6,341,741)	**Unadjusted:**<br>3,075,118 (2,371,693–3,788,135)<br><br>**Adjusted for underreporting:**<br>5,935,463 (4,497,316–7,377,636)	95% CI Uniform distribution

**Echinococcosiss: alveolar echinococcosis (*E. multilocularis*) and cystic echinococcosis (*E. granulosus*)**	2001-2003	China (Shiqu County)	1100	**Total losses** (excluding losses in calf production, carcass weight, and yak hide): 247 (214–279)<br><br>**Total losses** (including losses in calf production, carcass weight, and yak hide): 389 (342–438)	**Total losses** (excluding losses in calf production, carcass weight, and yak hide): 1347 (1314–1379)<br><br>**Total losses** (including losses in calf production, carcass weight, and yak hide): 1490 (1442– 1537)	95% CI Uniform distribution
**Cysticercosis (*Taenia solium*)**	2007	Mozambique (Angónia district)	2027 (1428–2761)	Without the proportion of pigs sold: 141 (81–230)<br><br>**Total:** 47 (27–76)	Without the proportion of pigs sold: 2173 (1569–2909)<br><br>**Total:** 2075 (1476–2809)	95 % UI Gamma distribution
**Cysticercosis (Taenia solium)**	2008	Cameroon	58987 (16,329–101,231)	568 (439–697)	59,540 (16,896–101,803)	95% CR Uniform distribution
**Cystic echinococcosis (E. granulosus)**	2010	Peru	1139	1099 (792–1407)	2238 (1931–2546)	95% CI Uniform distribution

**Rabies (Lyssavirus)**	2010	Worldwide	**Asia 2:** 368,376 (94,862–640,037)<br>**Asia 3:** 462097 (94,090–833,514)<br>**Asia 4:** 46619 (11,803–81,145)<br>**China:** 365023 (74,959–658,747)<br>**India:** 1,909,088 (453,985–3,358,527)<br>**Indonesia:** 105605 (16575–193418)<br>**North Africa:** 251,128 (48,721–455,977)<br>**Congo Basin:** 636,550 (263,527–1,011,627)<br>**West Africa:** 587,499 (224,634–952,020)	**Asia 2:** 420 (44–1611)<br>**Asia 3:** 87 (0.6–453)<br>**Asia 4:** 34 (6–207)<br>**China:** 1448 (405–2477)<br>**India:** 4580 (1439–7724)<br>**Indonesia:** 22 (0–506)<br>**North Africa:** 8 (0.5–73)<br>**Congo Basin:** 3 (0.3–36)<br>**West Africa:** 11 (0–186)<br>**SADC:** 5 (0–57)	**Asia 2:** 367,849 (94,900–641,049)<br>**Asia 3:** 464,757 (94,279–833,473)<br>**Asia 4:** 46485 (11,854–81,205)<br>**China**: 368536 (76,900–660,044)<br>**India:** 1907787 (457,488–3,364,968)<br>**Indonesia:** 105,310 (16,715–193,698)<br>**North Africa:** 253,229 (48,634–456,088)<br>**Congo Basin:** 638,791 (263,413–1,011,283)<br>**West Africa:** 587,641 (225,199–952,027)<br>**SADC:** 934,682 (196,022–1,674,590)	95% CI Uniform distribution, Poisson

			**SADC:** 939,689 (197,503–1,673,558)			
			**Andean:** 1994 (101–3898)	**Andean**: 2 (0.2–11)	**Andean:** 2009 (104–3905)	
			**Brazil:** 998 (50–1949)	**Brazil:** 3 (2–5)	**Brazil:** 1006 (52–1952)	
			**Caribbean:** 10459 (4308–16,672)	**Caribbean:** 0 (0–2)	**Caribbean:** 10,467 (4324–16,675)	
			**Central America:** 1493 (75–2925)	**Central:** 0.03 (0–5)	**Central America:** 1491 (74–2925)	
			**Southern Cone:** 503 (24–976)	**Southern Cone:** 0 (0–4)	**Southern Cone:** 500 (26–975)	
			**Eastern Europe:** 2497 (128–4875)	**Eastern Europe:** 0.12 (0 –2)	**Eastern Europe:** 2509 (126 –4874)	
			**Eurasia:** 206583 (54,047–359,951)	**Eurasia:** 5 (1–62)	**Eurasia:** 206,690 (54,015–360,086)	
			**Middle East:** 22,594 (6822–38,167)	**Middle East:** 0.15 (0.02–3)	**Middle East:** 22,532 (6848–38,182)	
			**Total:** 5,916,890 (1,544,600–10,282,026)	**Total:** 279 (101– 466)	**Total:** 5,920,014 (1,547,860–10,290,815)	

**Q fever** (***Coxiella burnetti***)	2007-2011	Netherlands	2833 (1071–4603)	2.86 (1.07–4.6)	2843 (1071–4603)	95% CI Uniform distribution
**Cysticercosis** (***Taenia solium***)	2012	Tanzania	30,443 (9264–72,115)	3985 (1485–6491)	34,455 (12,993–76,193)	95% UI Gamma distribution; Uniform distribution
**Rabies** (***Lyssavirus***)	2005-2014	Viet Nam	**Age 26:** 4956 (3432–6471); **Age 31:** 4450 (3086–5824); **Age 36:** 3955 (2744–5176)	3985 (1485 –6491)	**Age 26:** 5815 (4292–7331**); Age 31:** 5309 (3946–6683); **Age 36:** 4814 (3603–6035)<br>**Total:** 5316 (4382–6244)	95% CI Uniform distribution
**Rabies** (***Lyssavirus***)	2003-2015 Human data: 2007, 2010-2015	Kazakhstan	**Total:** 454 (339–593)<br>**Without PEP:** 7827 (4746–12074)	**Cattle:** 3 (2.8–3.25)<br>**Sheep:** 0.09 (0.07–0.11);<br>**Camel:** 0.016 (0.009 –0.03)<br>**Horse:** 0.3 (0.24 –0.42)<br><br>**Total:** 3.42 (3.16–3.7)	Cattle: 457 (342 –596)<br>Sheep: 454 (339 –594)<br>Camel: 454 (339–594)<br>Horse: 339 (454–594)<br>Total: 457 (342–597).<br><br>**Without PEP:**<br>Cattle: 7830 (4749–12,077)<br>Sheep: 7827 (4746–12,074)	95% CI Gamma distribution

					Camel: 7827 (4746–12,074)<br>Horse: 7827 (4746–12,076)<br>Total: 7831 (4749–12,077)	
**Brucellosis**<br>(***Brucella spp***)	2006-2015	Kazakhstan	713 (661–766)	1730 (1729–1731)	2443 (2391–2496)	95% CI<br>Poisson<br>distribution
**Leptospirosis**<br>(***Leptospira spp***)	2013-2019	New Zealand	**At risk of leptospirosis:** 14.07 (95% PI: 1.86–80.73)<br>**Not at risk of leptospirosis:** 3.69 (95% PI: 0.49–21.20)<br>**Total:** 17.76 (95% PI: 2.35–101.93)	178	**At risk of leptospirosis:** 192<br>**Not at risk of leptospirosis:** 182<br>Total: 196	----

**Brucella, Anthrax, Tularemia, CCHF, Rabies, Cystic Echinococcosis, Toxoplasmosis**	2016-2018	Turkey	Brucella: 1083 (818–1314)<br><br>Anthrax: 30 (0–135)<br><br>**Total** (Brucella, Anthrax, Tularemia, CCHF, Rabies, Cystic Echinococcosis, Toxoplasmosis): 1686 (1463–2207)	Brucella large ruminant: 1410 (840 –3324)<br><br>Brucella small ruminant: 265 (119–831)<br><br>Brucella total: 1675 (959-4155)<br>Anthrax large ruminant: 116 (97–240)<br>Anthrax small ruminant: 56 (46–111)<br>Anthrax total: 3176 (1103–7456)<br><br>**Total:** 1851 (1104–4500)	Brucella large ruminant: 2493 (1659–4637)<br><br>Brucella small ruminant: 1348 (937–2144)<br><br>Brucella total: 2758 (1778– 5467)<br><br>Anthrax large ruminant: 127 (116–375)<br>Anthrax small ruminant: 76 (56–246)<br><br>Anthrax total: 173 (166–486)<br><br>**Total:** 3538 (2567–6706)	95% CI Poisson distribution

The sum of values may not be exact since they are based on estimations randomly generated.

Most values are rounded to two significant figures

Four papers estimated the burden of rabies: Africa and Asia, Vietnam, Kazakhstan, and worldwide. The countries included in the worldwide study on rabies, Africa and Asia are listed in the supporting information pp 6-7. Viet Nam reported the DALYs by age (26, 31,36). Whereas Kazakhstan reported the values on rabies without post-exposure prophylaxis (PEP). The total zDALYs per capita was higher in Africa (11 zDALYs per 10,000 population) than Asia (3 zDALYs per 10,000 population).

Cystic echinococcosis ***(**E. granulosus**)*** was reported in Peru, Turkey, and on a global scale. In addition, a study in Shiqu County, China, studied both cystic echinococcosis, and alveolar echinococcosis (*E. multilocularis*).

For brucellosis, the Kazakh study only accounted for losses due to slaughtering of the animals and subsequent compensation. Whereas the Turkish study also considered reduced productivity. Besides, the Turkish study was the only one that included bacterial, parasitic, and viral zoonoses. However, we only determined the ALE for brucellosis and anthrax since the animal loss was only available for those diseases. We calculated the total zDALY for all the diseases included in this study.

Adding all the adjusted estimates for each study, we obtained a zDALY of 11,015,438 (95% CI: 6,235,971-15,806,100) and an ALE of 4,936,233 (95% CI: 3,512,616-6,357,435). The ALE represents approximately 45% of the total zDALYs.

Since the studies that already estimated zDALYs did not meet the inclusion criteria, we added their findings in the supporting information p 4.

Bias assessment – ROBIS

The full ROBIS assessment is provided in the supporting information pp 8-12. Overall, the risk of bias for this study is low. According to the signaling questions, there were no concerns regarding all the domains (study eligibility criteria, identification, selection of studies, and data collection). Therefore, the review is likely to include a high proportion of relevant studies.

However, the last domain (synthesis and findings) outlines that no meta-analysis was performed. We report the reasons in the discussion.

The PRISMA checklist is provided in the supporting information pp 13-15.

Discussion

We report the first systematic review that estimates the dual burden of zoonoses in humans and domestic animals based on studies available worldwide. Such information is needed for zoonosis prioritization, and resource allocation since interventions to control zoonoses are frequently carried out in animal hosts. Zoonoses impact health and socio-economic factors in multiple ways, increasing inequity between populations. Zoonoses in LICs are often under-reported compared to non-zoonotic diseases [29].

Despite the substantial burden caused by zoonoses in humans and animals, the number of studies combining both burdens is scarce. Besides, the use of old data does not reflect the current situation that depicts the dual burden of zoonoses. Studies that include human and animal data for zoonoses are relatively new (published in the last 20 years.) We observed an increased number of reports on the dual burden of diseases over the years. Up to date, only three studies have reported zDALYs: on cystic echinococcosis in Morocco[12], 25 zoonoses in Paraguay[10], *Taenia*

solium in Lao PDR.[11] We excluded them from our synthesis since they already contain zDALY values.

The dual burden of zoonoses was reported the most in Asia and Africa. The majority of zoonoses were based on estimations, due to the lack of reports, access to health care, and tools for disease diagnoses. The data source of the global estimates on rabies (Hampson et al.)[30] and the one reported in Asia and Africa (Knobel et al.)[16] have seven years difference. Both studies applied different ranges of uncertainty to their estimates and used different clusters. Therefore, comparing the zDALYs from Asia and Africa in both studies is slightly difficult. We report higher zDALYs for estimates from Hampson's study. If post-exposure prophylaxis is not considered, the burden increased by 5 times, because rabies is lethal, and hence the high DALYs contribute to higher zDALYs. Comparing the global rabies estimates provided by the Global Burden of Diseases (GBD)[31], and Hampson et al., the median of the latter was 2,665,145 DALYs more than the GBD's in 2010 (the year of the data source of Hampson et al. study.) However, the GBD estimated 2,529,389,250 DALYs more than Hampson's estimation for rabies in 2015 (year of publication of Hampson's study.)

Among diseases included in this review, echinococcosis was the most reported parasitic zoonosis. Cystic echinococcosis being the most common form reported. Echinococcosis causes a considerable burden because its treatment is expensive and complicated [32]. Alveolar echinococcosis *(E. multilocularis*) is considered rare worldwide, except for China, Russia, and the Kyrgyz Republic.[33,34] Alveolar echinococcosis (AE) rarely affects agricultural animals or pets (except for exceedingly rare cases of AE in dogs when they act as an intermediate host), so the health burden on animals is negligible. Dogs are common definitive hosts but do not show any

clinical symptoms. Cystic echinococcosis on a global scale was the only disease that had higher ALE compared to the DALY. Therefore, the animal burden had more influence on the total zDALYs of cystic echinococcosis worldwide. For the global estimation of cystic echinococcosis, *Budke et al* presented it as adjusted and unadjusted DALYs. They were higher than GBD's without exceptions (including period of data source and publication). The least difference was between the unadjusted values and GBD, mainly in 1996. For that year, the difference was 106,017 (with unadjusted values) and 833,436 (adjusted values). The unadjusted DALYs were similar to but higher than 285,000 DALY estimates for CE by the Foodborne Disease Burden Epidemiology Reference Group (FERG) – 184,000 DALYs [35]. This difference may be due to the lower disability weight (DW) used by FERG and GBD (abdominal discomfort) compared to Budke et al. (liver cancer). However, no specific DW has yet been developed for CE, so appropriate ones from diseases with similar morbidity have been used.

Cysticercosis was studied in three African countries. The highest zDALY on cysticercosis was calculated for Cameroon with data from 2008, followed by Tanzania (2012). However, Tanzania reported a higher ALE compared to Cameroon due to higher economic losses in the pig population. Mozambique data was only from the Agonia district; thus, the results are not comparable to the other countries. Although approximately only 0,9% of total zDALYs account for ALE in Cameroon, 2% in Mozambique, and 11% in Tanzania, respectively. When considering the zDALY per capita, Cameroon has the highest zDALY per capita (12 zDALYs per 1000 population), followed by Mozambique (6 zDALYs per 1000 population), and Tanzania (1 zDALY per 1000 population). Cameroon's cysticercosis estimated by Praet et al was higher than the GBD's. For cysticercosis in Tanzania, Trevisan's estimation was also higher than GBD's, being the least difference in 2017 (the

year of publication), around 24,166 DALYs. We assume the DALY on *T. solium* is higher than ALE, because it causes epilepsy in humans with high morbidity and mortality. Whereas the ALE on cysticercosis results only in organ condemnation. Furthermore, the lack of data on animals also contributes to a lower ALE. In Tanzania and Mozambique, these pigs lose half of their value, while in Cameroon the price usually is reduced by 30%. This demonstrates that cultural practices are relevant when estimating the impact or burden of a given condition on an animal population. It also shows, that the zDALY metric is able to represent such differences effectively.

Generally, the impact of zoonoses is usually associated with low- and middle-income countries. However, the studies in New Zealand and the Netherlands demonstrate that also high-income countries can suffer from losses in health, time, and money caused by zoonoses. Even though their impact is less than those in LICs and LMICs, they can worsen if appropriate preventive measures are not taken. For example, in the case of Q fever in the Netherlands, it was estimated that the loss of a culling milk goat is 100 times higher than a dose of the vaccine [22]. We estimated that in Netherlands Q-fever burden results to 2843 zDALYs, and only 2.86 is attributable to ALE. This could be because most of the infections due to *Coxiella burnetti* in animals are subclinical, and only result in abortions during late term. Furthermore, the control of Q-Fever is not included in these costs, however, authors mentioned that Q-fever control from the cost-utility perspective is expensive [22].

According to our findings, the burden of zoonoses impacts slightly more the human health sector, which is reflected in high DALYs rather than ALE, except for the estimations of the global cystic echinococcosis, leptospirosis in New Zealand, brucellosis in Kazakhstan, and zoonoses in Turkey (Figure 3). The total summed up estimates for our review resulted in 11,015,438 (95% CI:

6,235,971-15,806,100), with ALE representing almost half of the total zDALYs. However, it might be double counted for diseases such as rabies, and echinococcosis because estimates include both values for global burden and country specific burden.

Figure 3: Relative distribution of the DALYs and ALE among the studies

Excluded at the full-text screening stage (reasoning available in the supporting information), estimates provided by Roth et al.,[36] when converted to animal health benefits saved, result in the same ballpark ratio of DALY to ALE as our estimations for Kazakhstan and Turkey. Other studies (excluded from this review) with higher ALE than DALY were the 25 combined human and animal zoonoses in Paraguay [10] (zDALY), and cystic echinococcosis in Morocco [12]. They demonstrated how the priorities of countries on zoonoses can change if animal populations are taken into consideration. When countries have higher DALYs compared to ALE, the first question one must ask is whether this is due to a lack of data from the animal population or if it is because only losses to farmers due to animal zoonosis account for the ALE.

Our estimations are based on the results of previous studies which is a limitation of this study, besides the small number of papers. In some cases, the data available for humans and animals were not from the same period, reducing the accuracy of the estimations. Only three studies shared their code for the analysis (one of them partially), making the rest of the studies not reproducible. Also, the lack of availability of datasets following the FAIR principles did not allow us to obtain the confidence intervals of our choice. This shows the need for FAIR data application in the health area [37–39]. The lack of data continues to be a challenge, as the approach that is used to analyze it. We did not perform a meta-analysis due to the high variability among studies,

including the type of study, and analysis design. This is also evidence of a lack of standardized methods to unify the burden caused by zoonoses in humans and animals in the past, and the unfamiliarity of the existing metrics available for that aim.

The strength of this study consists of an extensive literature search in different databases without an initial time restriction. Considering that the GBD study does not include most of the zoonoses burden, as well as the animal burden of zoonosis, we integrated this data into the human burden among the studies available worldwide. The DALY is a metric used to prioritize international disease-control investments. However, its use has been debated for various, primarily ethical, reasons. Among which is a limited applicability to neglected tropical diseases (NTDs). Most NTDs in this study have a low chronic morbidity that accounts only for a small portion of DALY. In low-income settings, where poverty is dominant, this low morbidity raises little attention. Half of the world's hungry are subsistence farmers and rely heavily on agriculture for their livelihoods.[40] However, subsistence farming and hard physical work are common in those settings and the disabling effects of the NTDs are a main source of poverty. This circular causality cannot be captured through DALY calculations. The zDALY, at least, allows to include the burden from animal health losses, which are highly relevant in most poverty settings. How much subsistence farmers lose due to a zoonotic disease and how long it will take them to recover their losses should receive more attention in public health policy as it addresses an important determinant of human health.

Regarding vector-borne zoonoses, the only reported were tularemia and Crimean-Congo hemorrhagic fever (CCHF) in Turkey but without a direct association of their animal losses. We suggest establishing databases that incorporate human and animal diseases for each country, thus

on a global scale. For example, complement the GBD database with ALEs to move towards better integration of human and animal health policies.

A remaining challenge for the zDALY are animals without traded economic value. Therefore, other methods for estimating the ALE component of the zDALY (e.g., willingness to pay, pairwise comparisons or direct time trade off) in analogy to ecosystem services should be explored. Not only are more comprehensive metrics needed, but also a more integrative effort and support to face zoonosis in LICs and LMIC. For this endeavor, we consider the zDALY represents a step towards progress in zoonosis prioritization.

Acknowledgments

This study was partially supported by “Don Carlos Antonio Lopez” (BECAL) 7th/2019, and Section of Veterinary Epidemiology at the University of Zurich.

We thank Sabine Klein, the medical librarian, for assisting in the scientific publications search.

References

1. Taylor LH, Latham SM, Woolhouse MEJ. Risk factors for human disease emergence. Philos Trans R Soc B Biol Sci. 2001;356: 983–989. doi:10.1098/rstb.2001.0888

2. Mathews F. Chapter 8 Zoonoses in Wildlife: Integrating Ecology into Management. Adv Parasitol. 2009;68: 185–209. doi:10.1016/S0065-308X(08)00608-8

3. The FAO-OIE-WHO Collaboration Sharing responsibilities and coordinating global activities to address health risks at the animal-human-ecosystems interfaces. 2010 [cited 30 Apr 2022]. Available: https://www.oie.int/app/uploads/2021/03/final-concept-note-hanoi.pdf

4. George J, Häsler B, Mremi I, Sindato C, Mboera L, Rweyemamu M, et al. A systematic review on integration mechanisms in human and animal health surveillance systems with a view to addressing global health security threats. One Heal Outlook 2020 21. 2020;2: 1–15. doi:10.1186/S42522-020-00017-4

5. Bernstein AS, Ando AW, Loch-Temzelides T, Vale MM, Li B V., Li H, et al. The costs and benefits of primary prevention of zoonotic pandemics. Sci Adv. 2022;8: 4183. doi:10.1126/SCIADV.ABL4183/SUPPL_FILE/SCIADV.ABL4183_SM.PDF

6. Häsler B, Gilbert W, Jones A, Pfeiffer DU, Rushton J, Otte MJ, et al. The Economic Value of One Health in Relation to the Mitigation of Zoonotic Disease Risks. Curr Top Microbiol Immunol. 2012;365: 127–151. doi:10.1007/82_2012_239

7. Palmer SR. Oxford textbook of zoonoses : biology, clinical practice, and public health control. 2011; 884.

8. Disability-adjusted life years (DALYs). [cited 29 Apr 2022]. Available: https://www.who.int/data/gho/indicator-metadata-registry/imr-details/158

9. Torgerson PR, Ruegg S, Devleesschauwer B, Abela-Ridder B, Havelaar AH, Shaw APM, et al. zDALY: An adjusted indicator to estimate the burden of zoonotic diseases. One Heal. 2018;5: 40–45. Available: https://ovidsp.ovid.com/ovidweb.cgi?T=JS&CSC=Y&NEWS=N&PAGE=fulltext&D=pmnm4&AN=29911164 https://uzb.swisscovery.slsp.ch/openurl/41SLSP_UZB/41SLSP_UZB:UZB?sid=OVID:medline&id=pmid:29911164&id=doi:10.1016%2Fj.onehlt.2017.11.003&issn=2352-7714&isbn=&volume=5

10. Noguera LP, Rüegg S, Torgerson P. The burden of zoonoses in Paraguay: A systematic review. Petersen CA, editor. PLoS Negl Trop Dis. 2021;15: e0009909. doi:10.1371/JOURNAL.PNTD.0009909

11. Okello WO, Okello AL, Inthavong P, Tiemann T, Phengsivalouk A, Devleesschauwer B, et al. Improved methods to capture the total societal benefits of zoonotic disease control: Demonstrating the cost-effectiveness of an integrated control programme for Taenia solium, soil transmitted helminths and classical swine fever in northern Lao PDR. PLoS Neglected Trop Dis [electronic Resour. 2018;12: e0006782. Available:

https://ovidsp.ovid.com/ovidweb.cgi?T=JS&CSC=Y&NEWS=N&PAGE=fulltext&D=med15&AN=30231029 https://uzb.swisscovery.slsp.ch/openurl/41SLSP_UZB/41SLSP_UZB:UZB?sid=OVID:medline&id=pmid:30231029&id=doi:10.1371%2Fjournal.pntd.0006782&issn=1935-2727&isbn=&volume=1

12. Saadiid A, Amarir F, Filali H, Thys S, Rhalem A, Kirschvink N, et al. The socio-economic burden of cystic echinococcosis in morocco: A combination of estimation method. PLoS Negl Trop Dis. 2020;14: 1–20. doi:10.1371/journal.pntd.0008410

13. Moher D, Liberati A, Tetzlaff J, Altman DG, Altman D, Antes G, et al. Preferred reporting items for systematic reviews and meta-analyses: The PRISMA statement. PLoS Medicine. Public Library of Science; 2009. doi:10.1371/journal.pmed.1000097

14. Historical currency converter with official exchange rates from 1953. [cited 30 Apr 2022]. Available: https://fxtop.com/en/historical-currency-converter.php

15. ROBIS tool | Bristol Medical School: Population Health Sciences | University of Bristol. [cited 18 Jan 2022]. Available: http://www.bristol.ac.uk/population-health-sciences/projects/robis/robis-tool/

16. Knobel DL, Cleaveland S, Coleman PG, Fèvre EM, Meltzer MI, Miranda MEG, et al. Re-evaluating the burden of rabies in Africa and Asia. Bull World Health Organ. 2005;83: 360–368. Available: https://www.embase.com/search/results?subaction=viewrecord&id=L40704865&from=export

17. Budke CM, Deplazes P, Torgerson PR. Global socioeconomic impact of cystic echinococcosis. Emerg Infect Dis. 2006;12: 296–303. Available: https://ovidsp.ovid.com/ovidweb.cgi?T=JS&CSC=Y&NEWS=N&PAGE=fulltext&D=med6&AN=16494758 https://uzb.swisscovery.slsp.ch/openurl/41SLSP_UZB/41SLSP_UZB:UZB?sid=OVID:medline&id=pmid:16494758&id=doi:10.3201%2Feid1202.050499&issn=1080-6040&isbn=&volume=12&issue

18. Budke CM, Jiamin Q, Qian W, Torgerson PR. Economic effects of echinococcosis in a disease-endemic region of the Tibetan Plateau. Am J Trop Med Hyg. 2005;73: 2–10. doi:10.4269/ajtmh.2005.73.2

19. Trevisan C, Praet N, Pondja A, Assane YA, Dorny P, Magnussen P, et al. Assessment of the social burden of

Taenia solium Cysticercosis in Angónia District, Mozambique. Trop Med Int Heal. 2013;18: 109–110. doi:10.1111/tmi.12163

20. Praet N, Speybroeck N, Manzanedo R, Berkvens D, Nforninwe DN, Zoli A, et al. The disease burden of Taenia solium cysticercosis in Cameroon. PLoS Negl Trop Dis. 2009;3. doi:10.1371/journal.pntd.0000406

21. Hampson K, Coudeville L, Lembo T, Sambo M, Kieffer A, Attlan M, et al. Estimating the Global Burden of Endemic Canine Rabies. Carvalho MS, editor. PLoS Negl Trop Dis. 2015;9: e0003709. doi:10.1371/journal.pntd.0003709

22. van Asseldonk MA, Prins J, Bergevoet RH. Economic assessment of Q fever in the Netherlands. Prev Vet Med. 2013;112: 27–34. Available: https://ovidsp.ovid.com/ovidweb.cgi?T=JS&CSC=Y&NEWS=N&PAGE=fulltext&D=med10&AN=23866818 https://uzb.swisscovery.slsp.ch/openurl/41SLSP_UZB/41SLSP_UZB:UZB?sid=OVID:medline&id=pmid:23866818&id=doi:10.1016%2Fj.prevetmed.2013.06.002&issn=0167-5877&isbn=&volum

23. Trevisan C, Devleesschauwer B, Schmidt V, Winkler AS, Harrison W, Johansen M V. The societal cost of Taenia solium cysticercosis in Tanzania. Acta Trop. 2017;165: 141–154. Available: https://ovidsp.ovid.com/ovidweb.cgi?T=JS&CSC=Y&NEWS=N&PAGE=fulltext&D=med14&AN=26756713 https://uzb.swisscovery.slsp.ch/openurl/41SLSP_UZB/41SLSP_UZB:UZB?sid=OVID:medline&id=pmid:26756713&id=doi:10.1016%2Fj.actatropica.2015.12.021&issn=0001-706X&isbn=&vol

24. Shwiff SA, Brown VR, Dao TT, Elser J, Trung HX, Tien NN, et al. Estimating the economic impact of canine rabies to Viet Nam 2005-2014. PLoS Neglected Trop Dis [electronic Resour. 2018;12: e0006866. Available: https://ovidsp.ovid.com/ovidweb.cgi?T=JS&CSC=Y&NEWS=N&PAGE=fulltext&D=med15&AN=30307947 https://uzb.swisscovery.slsp.ch/openurl/41SLSP_UZB/41SLSP_UZB:UZB?sid=OVID:medline&id=pmid:30307947&id=doi:10.1371%2Fjournal.pntd.0006866&issn=1935-2727&isbn=&volume=1

25. Sultanov AA, Abdrakhmanov SK, Abdybekova AM, Karatayev BS, Torgerson PR. Rabies in Kazakhstan. PLoS Neglected Trop Dis [electronic Resour. 2016;10: e0004889. Available: https://ovidsp.ovid.com/ovidweb.cgi?T=JS&CSC=Y&NEWS=N&PAGE=fulltext&D=med13&AN=27486744

https://uzb.swisscovery.slsp.ch/openurl/41SLSP_UZB/41SLSP_UZB:UZB?sid=OVID:medline&id=pmid:27486744&id=doi:10.1371%2Fjournal.pntd.0004889&issn=1935-2727&isbn=&volume=1

26. Charypkhan D, Sultanov AA, Ivanov NP, Baramova SA, Taitubayev MK, Torgerson PR. Economic and health burden of brucellosis in Kazakhstan. Zoonoses Public Heal. 2019;66: 487–494. Available: https://ovidsp.ovid.com/ovidweb.cgi?T=JS&CSC=Y&NEWS=N&PAGE=fulltext&D=med16&AN=31090193 https://uzb.swisscovery.slsp.ch/openurl/41SLSP_UZB/41SLSP_UZB:UZB?sid=OVID:medline&id=pmid:31090193&id=doi:10.1111%2Fzph.12582&issn=1863-1959&isbn=&volume=66&issue=5&s

27. Sanhueza JM, Baker MG, Benschop J, Collins-Emerson JM, Wilson PR, Heuer C. Estimation of the burden of leptospirosis in New Zealand. Zoonoses Public Heal. 2020;67: 167–176. Available: https://ovidsp.ovid.com/ovidweb.cgi?T=JS&CSC=Y&NEWS=N&PAGE=fulltext&D=med18&AN=31799801 https://uzb.swisscovery.slsp.ch/openurl/41SLSP_UZB/41SLSP_UZB:UZB?sid=OVID:medline&id=pmid:31799801&id=doi:10.1111%2Fzph.12668&issn=1863-1959&isbn=&volume=67&issue=2&s

28. ARI HO, İŞLEK E, BİLİR MK, ... The monetary impact of zoonotic diseases on society: The Turkish Case Study. Ankara …. 2022. Available: http://vetjournal.ankara.edu.tr/en/pub/auvfd/issue/48904/789598

29. Schelling E, Grace D, Willingham AL, Randolph T. Research Approaches for Improved Pro-Poor Control of Zoonoses. Food Nutr Bull. 2007;28: S345–S356. doi:10.1177/15648265070282S214

30. Hampson K, Coudeville L, Lembo T, Sambo M, Kieffer A, Attlan M, et al. Estimating the Global Burden of Endemic Canine Rabies. PLoS Negl Trop Dis. 2015;9. doi:10.1371/journal.pntd.0003709

31. GBD Results Tool | GHDx. [cited 28 Jun 2020]. Available: http://ghdx.healthdata.org/gbd-results-tool

32. Echinococcosis. [cited 5 Apr 2022]. Available: https://www.who.int/news-room/fact-sheets/detail/echinococcosis

33. Torgerson PR, Keller K, Magnotta M, Ragland N. The Global Burden of Alveolar Echinococcosis. PLoS Negl Trop Dis. 2010;4: e722. doi:10.1371/JOURNAL.PNTD.0000722

34. Paternoster G, Boo G, Wang C, Minbaeva G, Usubalieva J, Raimkulov KM, et al. Epidemic cystic and alveolar

echinococcosis in Kyrgyzstan: an analysis of national surveillance data. Lancet Glob Heal. 2020;8: e603–e611. doi:10.1016/S2214-109X(20)30038-3

35. Torgerson PR, Devleesschauwer B, Praet N, Speybroeck N, Willingham AL, Kasuga F, et al. World Health Organization Estimates of the Global and Regional Disease Burden of 11 Foodborne Parasitic Diseases, 2010: A Data Synthesis. PLOS Med. 2015;12: e1001920. doi:10.1371/JOURNAL.PMED.1001920

36. Roth F, Zinsstag J, Orkhon D, Chimed-Ochir G, Hutton G, Cosivi O, et al. Human health benefits from livestock vaccination for brucellosis: case study. Bull World Health Organ. 2003;81: 867–876. Available: https://ovidsp.ovid.com/ovidweb.cgi?T=JS&CSC=Y&NEWS=N&PAGE=fulltext&D=med5&AN=14997239 https://uzb.swisscovery.slsp.ch/openurl/41SLSP_UZB/41SLSP_UZB:UZB?sid=OVID:medline&id=pmid:14997239&id=doi:&issn=0042-9686&isbn=&volume=81&issue=12&spage=867&pages=867-

37. Wilkinson MD, Dumontier M, Aalbersberg IjJ, Appleton G, Axton M, Baak A, et al. The FAIR Guiding Principles for scientific data management and stewardship. Sci Data 2016 31. 2016;3: 1–9. doi:10.1038/sdata.2016.18

38. FAIR Principles - GO FAIR. [cited 25 Apr 2022]. Available: https://www.go-fair.org/fair-principles/

39. Meyer A, Faverjon C, Hostens M, Stegeman A, Cameron A. Systematic review of the status of veterinary epidemiological research in two species regarding the FAIR guiding principles. BMC Vet Res. 2021;17: 1–14. doi:10.1186/S12917-021-02971-1/TABLES/4

40. Final study of the Human Rights Council Advisory Committee on the advancement of the rights of peasants and other people working in rural areas /. [cited 10 May 2022]. Available: https://digitallibrary.un.org/record/720467

Supporting information

Pages 1-2, Supporting Information 1, List of used terms for each electronic search

Page 3, Supporting Information 2, List of papers excluded at the full-text screening, with reasons of exclusion

Page 4, Supporting Information 3, Papers with zDALYs estimation excluded from this review

Page 5, Supporting Information 4, List of included papers

Pages 6-7, Supporting Information 5, List of countries included in the rabies studies (at global and continental levels)

Pages 8-11, Supporting Information 6, ROBIS tool

Page 12-14, Supporting Information 7, PRISMA checklist

Page 15, Supporting Information 8, Figures

Figure1 Click here to access/download;Figure;Fig1.tif

Pasted Layer

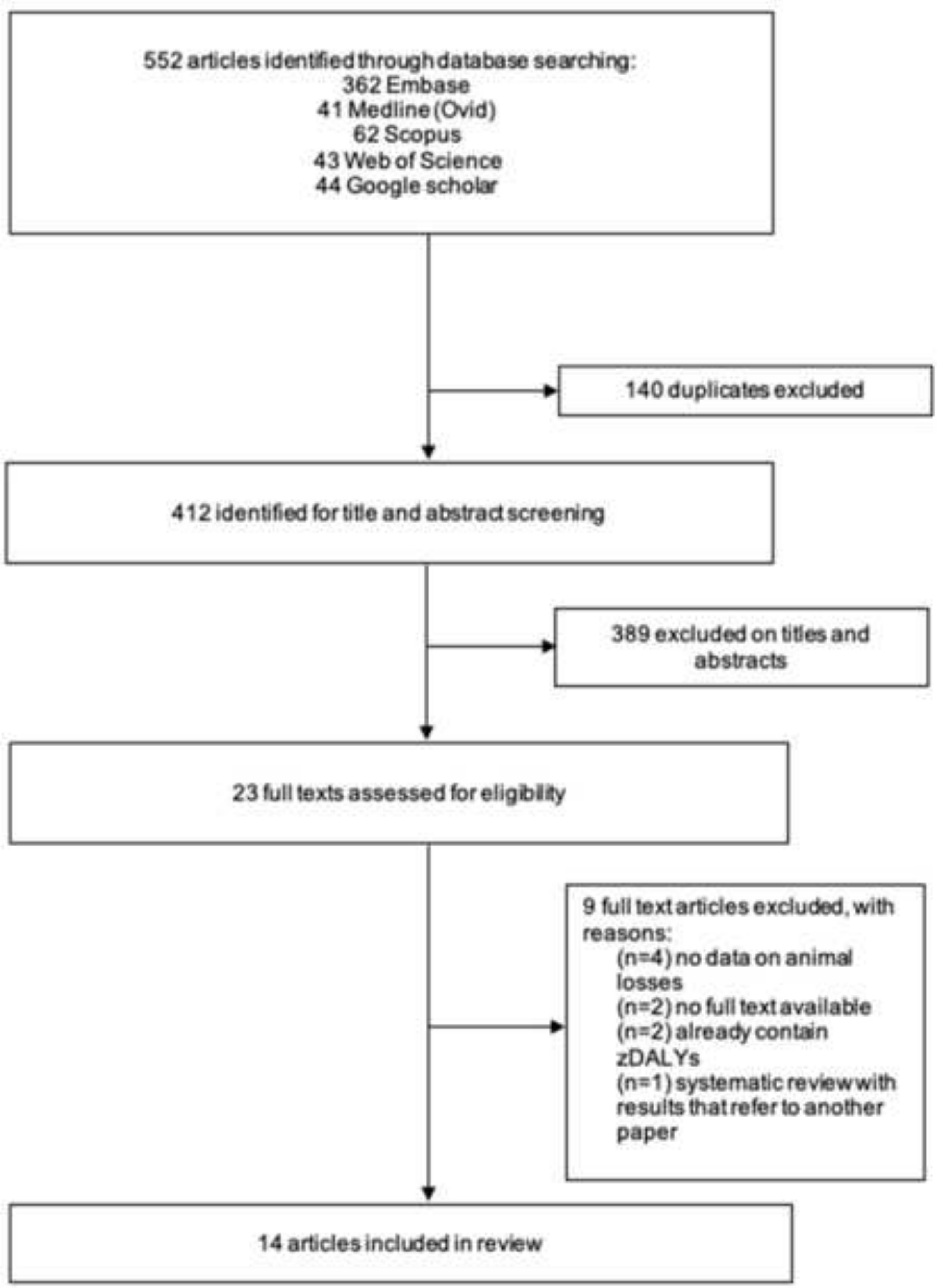

Figure2

Click here to access/download;Figure;Fig2.tif

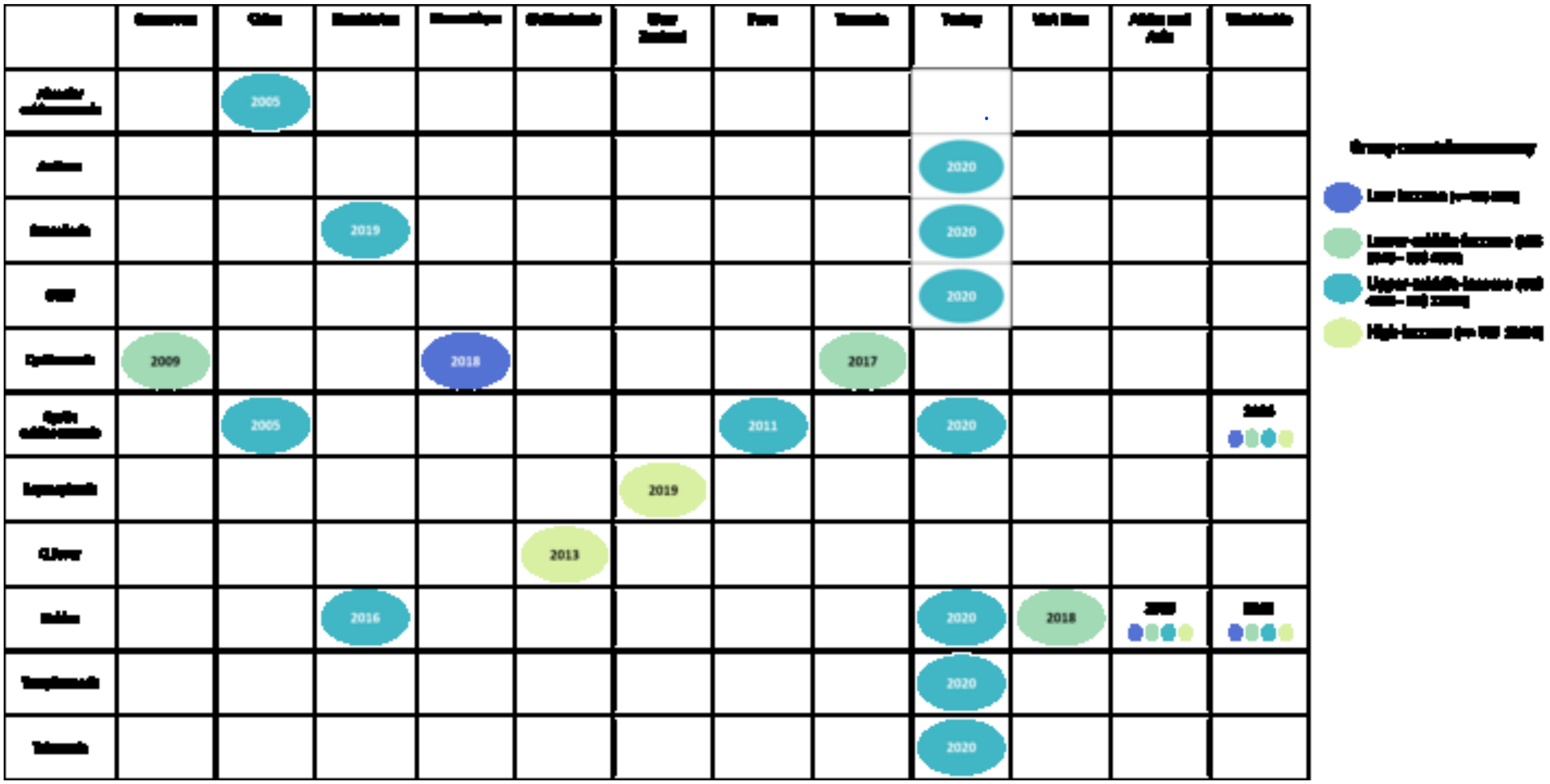

Pasted Layer

Figure3

Click here to access/download;Figure;Fig3.tiff

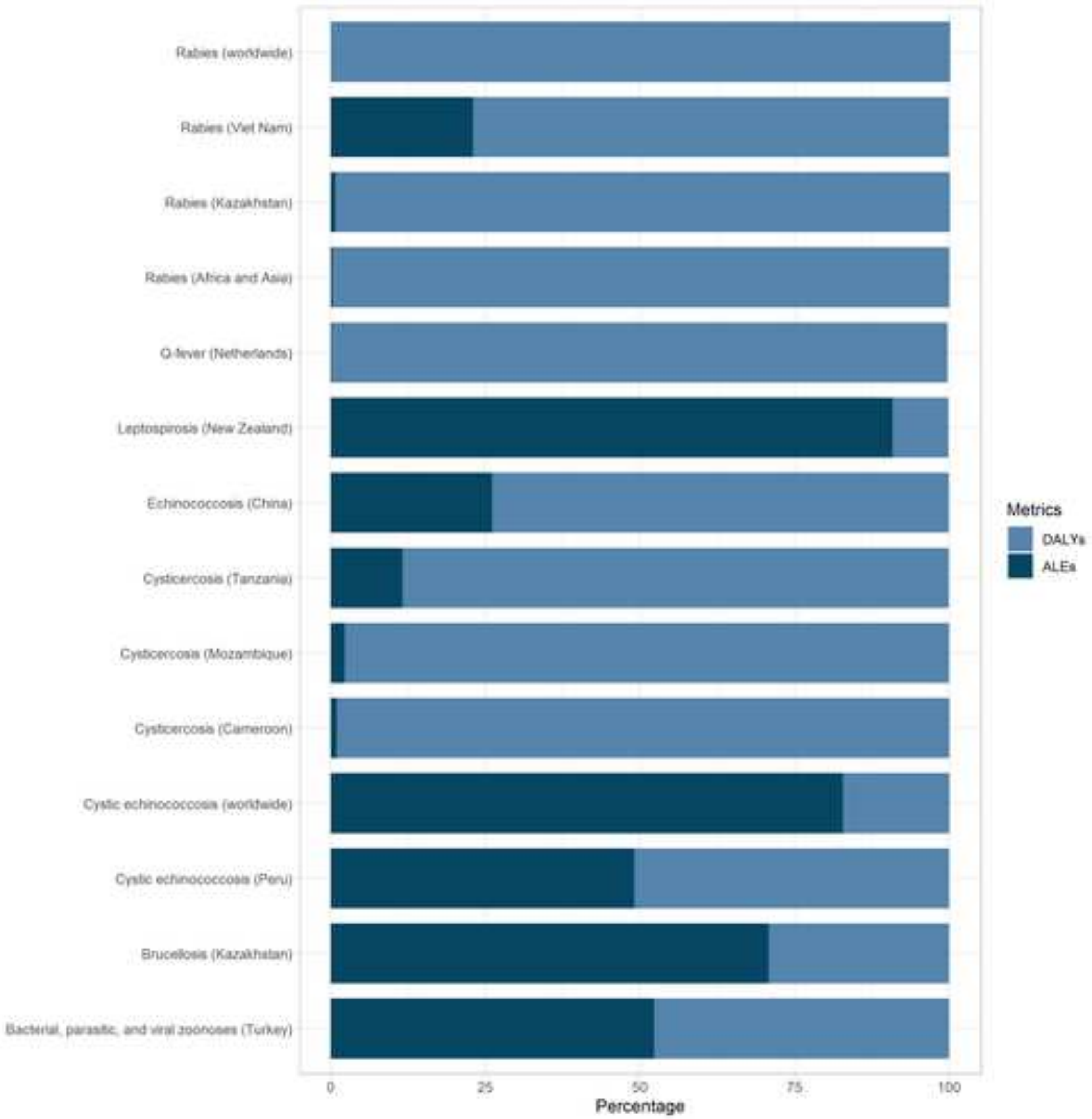

SI

Click here to access/download
Supporting Information
Supporting information_dual burden Plos NTD.docx
