# Supplementary material for: The dual burden of animal and human zoonoses: A systematic review
Source: PLoS Negl Trop Dis. 2022 Oct 14;16(10):e0010540. doi: 10.1371/journal.pntd.0010540 (PMC9605338; doi:10.1371/journal.pntd.0010540)
Supplement: Attachment — Submitted filename: Response to the reviewers.docx [file pntd.0010540.s013.docx]

**Manuscript ID:** PNTD-D-22-0069

**Manuscript title:** The dual burden of animal and human zoonoses: a systematic review.

Dear Editors,

We thank the reviewers for their time and helpful comments. We have addressed their concerns to improve the manuscript.

The two most significant revisions to address are:
 1) Clarification of some of the sampling and statistical techniques you used.
 2) Figure 2 is of very poor quality and essentially unreadable - please replace this with a figure of higher quality for your re-submission.

**Response:** Although we address the reviewers' comments point by point below, we would also like to outline here that we did not perform a meta-analysis, thus the pooling of the data was not performed. Therefore, we cannot report the heterogeneity methods and/or results. Otherwise, it is not clear to us what exactly the reviewer requires and would seek further clarifications. We only report the results of previous studies, extracting data, then analyzing it from each selected publication to finally report it. We could assume that this led possibly to confusion because we reported the total values for zDALYs, which is actually not correct from our side. Thus, in order not to confuse the readers, and the reviewer, we refrain from reporting the total zDALY values in our manuscript, since we are not doing the global estimates, but demonstrating a methodology to get the dual burden of zoonoses.

Regarding the figures included in this manuscript, we enhanced the quality and ensured that they now meet the journal requirements (300 DPI resolution).

**Methods**

**Reviewer #3:**
-Were correct statistical analysis used to support conclusions?
 No. I think statistical methods needs more clarification.
 -Are there concerns about ethical or regulatory requirements being met?
 No

**Response:** It is not clear what exactly the reviewer requires as we were not pooling results or making population estimates based on a representative number of studies. We reported the results of a systematic review, extracting data then analyzing it from each selected publication to finally report it. We also shared the code in git so that the study can be reproducible. Although we did not perform a metanalysis (quantitative synthesis), we synthesized the results through tables and graphs presented in this systematic review which was added to the PRISMA guidelines checklist.

We added one more paragraph to the methodology (lines 121-124 track changes version). For the data distributions and the confidence intervals, we fitted the data according to what previous authors did (so, we reproduced what they described in the methodology and results). Thus, we used the lower and upper bounds reported in the confidence intervals to reproduce the DALYs, and animal loss. The distributions varied according to the data available, and the description in the methodology of previous studies. Then, we estimated the Animal Loss Equivalent (ALE = annual monetary value of animal health losses/ GNI per capita in US$ at the period of the study). Finally, to sum up the DALY + ALE = zDALY. Based on the estimation of zDALYs, we computed the confidence intervals, which are described in the methodology, and further details are available at <https://github.com/LizPNZ/Dual-burden-of-zoonosis>

We corrected the values reported as total zDALYs. Instead, we refrain from reporting the total zDALY values in our manuscript, since we are not doing the global estimates, but demonstrating a methodology to get the dual burden of zoonoses.

Regarding the bias assessment, we performed it qualitatively through ROBIS tool, added to the supplementary information.

--------------------

**Results**

-Does the analysis presented match the analysis plan?

-Are the results clearly and completely presented?

-Are the figures (Tables, Images) of sufficient quality for clarity?

**Reviewer #1:** (No Response)

**Reviewer #2:** Yes.

**Reviewer #3:** -Does the analysis presented match the analysis plan?
No. the used methods for pooling data is not clear. heterogenicity methods and results of them is not reported.

**Response:** We would like to clarify that our estimations are based on the data extracted from studies that we included in the systematic review. Based on your comments, we decided to report the zDALY by studies to better convey the methodology in order to get a dual estimate of the burden of zoonotic diseases.

-Are the results clearly and completely presented? Tables are complex with some undefined headers.

***Response:*** *Thank you for the comment. We added headers and improved the tables.*

-Are the figures (Tables, Images) of sufficient quality for clarity? The quality of figures was too low and I was unable too assess them.

***Response:*** *The figures were improved and are now of quality of 300 DPI.*

--------------------
 **Editorial and Data Presentation Modifications?**

Use this section for editorial suggestions as well as relatively minor modifications of existing data that would enhance clarity. If the only modifications needed are minor and/or editorial, you may wish to recommend “Minor Revision” or “Accept”.

**Reviewer #1:** (No Response).

**Reviewer #2:** I suggest improving the quality of the figures, especially figure 2.

***Response:*** *The figures were improved and now comply with the PLOS NTD requirements and are of quality of 300 DPI.*

**Reviewer #3:** Dear editor

The topic of this study is attractive, and I think it would be improved using reviewers comment. I recommended major revision to revise the statistical methods and results.

***Response:*** *we addressed the comments and suggestions provided by the reviewers point by point in the rebuttal letter and in the manuscript.*

--------------------

**Summary and General Comments**

Use this section to provide overall comments, discuss strengths/weaknesses of the study, novelty, significance, general execution and scholarship. You may also include additional comments for the author, including concerns about dual publication, research ethics, or publication ethics. If requesting major revision, please articulate the new experiments that are needed.

**Reviewer #1:** This study presents the first systematic review that estimates the dual burden of zoonoses in humans and domestic animals based on studies available worldwide. This is an interesting study, which provides important information for the prioritization of the control and prevention of zoonotic diseases. Please see below my comments and suggestions.

***Response:*** *Thank you for your suggestions. We addressed them point by point below.*

**ABSTRACT**
Lines 25-27: I suggest rephrasing this sentence as it is currently unclear whether the ALE are for rabies and echinococcosis only.

***Response:*** *We improved the abstract.*

**INTRODUCTION**
Line 63: I suggest adding a definition of Disability Adjusted Life Years (DALY).

***Response:*** *We added the DALY definition: “*The DALYs consist of the loss of health due to a disease (or disability) and premature mortality”.

**METHODS**
Line 91: Did you include studies written in a language other than English?

I am concerned that your search strategy missed relevant studies:
-	Did you consider doing a reference list search of studies included for full-text review or in the review for potential additional studies?

-	To what extent do you think using the term “zoonoses” rather than the actual zoonotic disease names in your literature search (i.e., rabies, echinococcosis, cysticercosis, brucellosis, leptospirosis, etc.), decreased the number of studies identified?

***Response:*** *Thank you for your question. The librarian and researchers tried out different combinations of search terms, including the strings that incorporate the actual disease's name. The yield was not different than when using the “zoonoses.” Regarding the language, the search was not restricted specifically to English. The librarian included terms that were common in different languages for some strings. For example, the term “animal” which is valid for Spanish, French, Portuguese, English, among others; or the string “zoono” which has a Greek root and is common in different languages.*

**RESULTS**
Line 160: Replace “e.g.” with “for example”.

***Response:*** *The suggestion was applied in the manuscript.*

**DISCUSSION**
 While it is clear why the three studies already including zDALY estimates were excluded from the actual review, it would have been relevant to include them in the discussion section, and discuss their results more extensively (e.g., include them in an overall total summed up estimate of zDALY, etc.).

***Response:*** *We added a paragraph to the discussion about this suggestion.*

*Line 312-316 (in track changes version): “The excluded studies with zDALYs were neurocysticercosis in Northern Lao PDR with 3497 zDALYs, cystic echinococcosis in Morocco 18,330 (95% CI 17,775–19,074), and (bacterial, parasitic, viral, fungal) zoonoses in Paraguay with zDALYs of 62,178 (95% CI 48,696-77,188) (SI 3). The percentages attributable to animal burden were 0.4% for Northern Lao PDR, 99% for Morocco, and 69% for Paraguay”*

Line 293: You mention “the lack of availability of datasets following the FAIR principles did not allow us to obtain the confidence intervals of our choice”; did you try contacting the corresponding authors of these studies to obtain the data? Same question for the studies that did not share their code.

***Response:*** *Thank you for your comment. We have not contacted the authors to try accessing the data or code due to time restriction.*

Line 314: Which determinant of human health?

***Response:*** *We added to the text, “social and economic environment**.”*

*“How much subsistence farmers lose due to a zoonotic disease and how long it will take them to recover their losses should receive more attention in public health policy as it addresses an important determinant of human health which mainly consists of the social and economic environment”*

**OTHER**
 The use of “Vietnam” or “Viet Nam” should be consistent throughout the manuscript.
 Please check the consistency in the references – sometimes they are cited after the sentence (e.g., “.[8]” instead of “[8].”).

Abbreviations should be defined only once at first use, and not multiple times throughout the manuscript (e.g., ALE and zDALY). Additionally, all abbreviations should be defined (e.g., LIC and LMIC are not).

***Response:*** *The suggestions were applied to the manuscript.*

Reviewer #2: The article presents a systematic review that addresses an innovative theme within the concept of One Health. The review was carried out with adequate methodological rigor.

Reviewer #3: Dear author thanks for your great study. I think you chose great topic, but methods and results section needs more clarification; you didn't explain well about statistical methods (pooling the results method, heterogenicity assessment method, ...) and I think tables are complex, please see my comments in you article PDF. your claim in PRISMA checklist was not according pages of PDF.

***Response:*** *Thank you for the comments. We addressed the comment about statistical methods above.*

*Regarding the PRISMA checklist, we corrected the pages. However, it might change after the edition. In some cases, we preferred to add the subsections of the manuscript instead of pages.*

--------------------

PLOS authors have the option to publish the peer review history of their article ( <https://journals.plos.org/plosntds/s/editorial-and-peer-review-process#loc-peer-review-history>" what does this mean?. If published, this will include your full peer review and any attached files.
If you choose “no”, your identity will remain anonymous, but your review may still be made public.

Do you want your identity to be public for this peer review? For information about this choice, including consent withdrawal, please see our "<https://www.plos.org/privacy-policy>" Privacy Policy

Reviewer #1: No

Reviewer #2: No

Reviewer #3: No
